# G3BP1 Interact with JAK2 mRNA to Promote the Malignant Progression of Nasopharyngeal Carcinoma via Activating JAK2/STAT3 Signaling Pathway

**DOI:** 10.7150/ijbs.85341

**Published:** 2024-01-01

**Authors:** Yuting Zhan, Weiyuan Wang, Haihua Wang, Yue Xu, Yuting Zhang, Yue Ning, Hongmei Zheng, Jiadi Luo, Yang Yang, Hongjing Zang, Ming Zhou, Songqing Fan

**Affiliations:** 1 Department of Pathology, the Second Xiangya Hospital, Central South University, Changsha, Hunan, China.; 2 Department of Pathology, Xiangya Hospital of Central South University, Changsha, Hunan, China.; 3 Cancer Research Institute and School of Basic Medicine Sciences, Central South University, Changsha, Hunan, China.

**Keywords:** G3BP1, RNA binding protein, JAK2/STAT3 signaling pathway, malignant progression

## Abstract

Ras-GTPase-activating protein (GAP)-binding protein 1 (G3BP1) is an RNA-binding protein implicated in various malignancies. However, its role in nasopharyngeal carcinoma (NPC) remains elusive. This study elucidates the potential regulation mechanisms of G3BP1 and its significance in NPC advancement. Through knockdown and overexpression approaches, we validate G3BP1's oncogenic role by promoting proliferation, migration, and invasion *in vitro* and *in vivo*. Moreover, G3BP1 emerges as a key regulator of the JAK2/STAT3 signaling pathway, augmenting JAK2 expression via mRNA binding. Notably, epigallocatechin gallate (EGCG), a green tea-derived antioxidant, counteracts G3BP1-mediated pathway activation. Clinical analysis reveals heightened G3BP1, JAK2, and p-STAT3 as powerful prognostic markers, with G3BP1's expression standing as an independent indicator of poorer outcomes for NPC patients. In conclusion, the study unveils the oncogenic prowess of G3BP1, its orchestration of the JAK2/STAT3 signaling pathway, and its pivotal role in NPC progression.

## Introduction

Nasopharyngeal carcinoma (NPC), an epithelial malignancy characterized by heterogeneity, predominantly afflicts regions in southern China, Southeast Asia, and North Africa 1. Notably, Epstein-Barr virus (EBV) infection underpins non-keratinizing nasopharyngeal carcinoma in South China, implicating a viral etiology 2, 3. With approximately 64,165 novel cases of NPC projected for diagnosis and 36,315 associated fatalities according to China's cancer statistics for 2022 4, the urgency to comprehend its pathogenesis remains paramount. A nexus of factors including EBV infection, aberrant oncogenic signaling, and oncogene overexpression has been implicated in NPC etiology 5, 6. Despite strides in early detection and local management, a subset of NPC patients succumbs to distant metastasis, engendering a grim prognosis.

Ras-GTPase-activating protein (GAP)-binding protein 1 (G3BP1), recognized for its multifaceted RNA-binding functionality, emerges as a pivotal orchestrator in stress granule (SG) assembly and dynamics 7. Our previous study unveiled elevated mRNA and protein levels of G3BP1 in non-small cell lung cancer (NSCLC) tissues relative to adjacent normal counterparts. Distinctly, patients in clinical stage II and III exhibited augmented G3BP1 expression compared to stage I counterparts. Moreover, overexpression of G3BP1 protein correlated with unfavorable prognosis in NSCLC patients independently 8. Noteworthy implications of G3BP1 extend to breast, colon, esophageal, and gastric cancer 9-12. In our precedent work, G3BP1's association with SGs came to light, triggered by specific stimuli and co-localizing with YB1 13. However, G3BP1's involvement in nasopharyngeal carcinoma, delinked from SGs formation, remains an unexplored terrain warranting investigation.

The Janus kinase-signal transducer and activator of transcription (JAK/STAT) signaling pathway assumes a pivotal role in an array of physiological and pathological processes, encompassing cell proliferation, differentiation, apoptosis, angiogenesis, immunosuppression, and sustained inflammatory responses 14, 15. However, the activation and regulation of this complex pathway remain intricate. Following ligand-receptor interaction, JAKs experience transphosphorylation, subsequently phosphorylating signal transducers and activators of STATs. This phosphorylation cascade culminates in the formation of homodimers or heterodimers, which then translocate to target gene promoters, instigating the orchestration of gene transcription 16. The repertoire of target genes spans diverse biological activities, as well as seemingly disparate functions like cell cycle regulation, apoptosis, and epithelial-mesenchymal transition (EMT) 17-20. Significantly, JAK/STAT signaling emerges as an overarching participant in the emergence and progression of various malignancies, including NPC 21, 22. Numerous factors impinge upon JAK/STAT signaling dynamics, encompassing cytokines, non-coding RNA, and protein expression. Particularly noteworthy among these factors are RNA binding proteins (RBPs). For instance, the re-initiation and release factor (DENR) interplays with and governs JAK2, thereby orchestrating the JAK2/STAT3 signaling axis 23. The pantheon of RBPs is believed to collectively shape the regulation of JAK/STAT signaling, ushering in the concept of the RBP-JAK/STAT network as a pioneering avenue for comprehending the intricate mechanisms underlying the pathogenesis of malignant tumors 24-26.

To unravel the plausible implication of G3BP1 in JAK/STAT signaling activation within the context of NPC, we embarked on a comprehensive investigation. Our approach encompassed an exhaustive scrutiny of G3BP1 mRNA expression across diverse malignant malignancies, NPC included, exploiting the resources of TCGA and GEO databases. Moreover, we assessed the clinical relevance of G3BP1 across these malignancies. Concurrently, our inquiry extended to discern the repercussions of G3BP1 on NPC cell proliferation and migration, meticulously assessed through *in vitro* experiments. Through rigorous investigation, we unearthed G3BP1's capacity to activate the JAK2/STAT3 signaling pathway by directly binding to JAK2 mRNA. Fascinatingly, this regulatory interaction was found susceptible to disruption upon treatment with Epigallocatechin gallate (EGCG), a potent antioxidant present in green tea. Employing subcutaneous tumorigenesis in nude mice and a metastatic tumor model, we incontrovertibly demonstrated G3BP1's contributory role in promoting proliferation and migration *in vivo*. The culmination of our efforts encompassed a meticulous analysis of G3BP1 expression in NPC and control nasopharyngeal epithelial tissues by immunohistochemistry (IHC). This endeavor was undertaken to ascertain the relationship between G3BP1 and clinicopathological attributes, as well as to unravel its potential prognostic significance in NPC patients. Significantly, this study marks the pioneering unveiling of G3BP1's involvement in JAK/STAT signaling activation within the context of NPC, independent of its hitherto linked role with SGs.

## Material and Methods

### Ethics statement

The study meticulously adhered to established ethical guidelines, securing informed consent from all participants or their legal guardians. Approval for the protocols, specimen utilization, and data retrieval was conferred by the Ethics Review Committee of the Second Xiangya Hospital of Central South University (Scientific and Research Ethics Committee, No. K022/2021). Consent forms, duly signed by all adult participants, along with appropriate documentation obtained from legal guardians, caretakers, or guardians for minors, served as a testament to the informed consent process. Every experimental undertaking meticulously aligned with pertinent guidelines and regulations governing ethical research conduct.

### Bioinformatics databases

To bolster the substantiation of our research findings, this investigation harnessed an array of bioinformatics databases and tools, thereby augmenting the comprehensiveness of our study. The Cancer Genome Atlas (TCGA) database (https://portal.gdc.cancer.gov/) emerged as a pivotal resource, enabling the scrutiny of G3BP1 mRNA expression across an expansive spectrum of 33 malignant tumor types, coupled with their corresponding unpaired tissues. Furthermore, within the realm of paired tissues, the study encompassed 18 pairs of malignant and normal tissues, contributing to a comprehensive analysis. Similarly, the Gene Expression Omnibus (GEO) database (http://www.ncbi.nlm.nih.gov/geo/) emerged as a crucial arsenal, specifically drawing from Dataset GSE12452. This dataset facilitated an incisive evaluation of G3BP1 mRNA expressions within the precincts of NPC and its juxtaposed adjacent normal control tissues.

Intricacies of protein-RNA interaction dynamics were probed through the RNAct database (http://rnact.crg.eu), which aptly predicted the intricate connections linking G3BP1 with its cognate RNA partners 27. Additionally, the exploration extended to RBPsuite (http://www.csbio.sjtu.edu.cn/bioinf/RBPsuite/), serving as a versatile predictive tool to anticipate the potential binding sites shared between G3BP1 and JAKs 28. Intriguingly, the investigation plumbed into molecular interactions through Autodock Vina V1.2.2, an established computational tool facilitating the prediction of binding affinities. This utility facilitated the forecast of feasible binding interactions encompassing EGCG and G3BP1, underpinned by visible hydrogen bonding and robust electrostatic interactions 29. The collective utilization of these databases and tools bestowed robust analytical underpinnings, thereby amplifying the rigor of our investigative pursuits.

### Cell lines, cell culture, and virus packaging

The immortalized nasopharyngeal epithelial cell line (NP69) and an array of human NPC cell lines (CNE1, CNE2, HNE1, HNE2, 5-8F, 6-10B, HK1, and HONE1) were generously bestowed by the Cancer Research Institute and School of Basic Medicine Sciences, Central South University. These cell lines underwent thorough validation via short tandem repeat (STR) profiling, a robust authentication process conducted by Microread Gene Technology (Beijing, China). This authentication procedure underscored the fidelity of the cell line identities. In accordance with these established procedures, cells were nurtured under optimal conditions to ensure their vitality and physiological state. To generate cells with augmented G3BP1 expression, lentiviral infection was harnessed. In this regard, the G3BP1-overexpressing lentivirus was procured from GenePharma (Suzhou, China). This lentiviral infection strategy emerged as an effective modality for endowing cells with heightened G3BP1 expression levels.

### Small interfering RNAs, plasmids, and transfection

To orchestrate effective gene silencing, small interfering RNAs (siRNAs) constituted a fundamental tool, collectively with the control siRNA (siNC), which served as a baseline comparator. The specific siRNAs (RiboBio Technology, Guangzhou, China) utilized encompassed siG3BP1-1 (CATTAACAGTGGTGGGAAA), siG3BP1-2 (AGGCTTTGAGGAGATTCAT), siJAK2-1 (GCAGAATTAGCAAACCTTATA) and siJAK2-2 (GCTTTGTCTTTCGTGTCATTA), targeting distinct domains of G3BP1 and JAK2, respectively. Meanwhile, the sequence for the control siRNA (siNC) remained proprietary and confidential, in accordance with the provider's protocol. Additionally, the study enlisted the G3BP1 and JAK2 plasmid housed within the vector pCMV3-N-HA. Concomitantly, the vector employed was the standardized pCMV3-N-HA vector, universally recognized within the research community (Sino Biology, Beijing, China).

### CCK8 assay, colony formation, wound healing assay, and Matrigel invasion assay

The CCK-8 assay, colony formation, wound healing assay, and Matrigel invasion assay were meticulously executed in consonance with the protocol stipulated in our antecedent study 13. These methodologies facilitated the systematic assessment of diverse cellular attributes, serving as a continuum of the investigative process.

### Cell cycle analysis by flow cytometry

Upon siRNA or plasmid transfection spanning a 24-hour timeframe, cells were subsequently transferred into 6 cm wells and nurtured for predefined intervals. The ensuing PI/RNAse cell cycle assay ensued as per the manufacturer's guidelines (C1052; Beyotime, China). Flow cytometry played a pivotal role in the scrutiny of stained cells, furnishing a detailed portrayal of cell cycle dynamics.

### Apoptosis analysis by flow cytometry

Employing the prowess of flow cytometry, cell apoptosis quantification emerged as a robust investigative facet. Following a 48-hour transfection interval, cells underwent harvesting and were subjected to PBS washing. The ensuing staining with PI/FITC-Annexin V (Zenbio, China), followed by a 10-minute incubation in darkness at 37°C, paved the way for flow cytometry-driven quantification of cell apoptosis percentage.

### Spheroid 3D invasion assay

Adhering to well-established protocols as documented in prior research 30, the execution of the spheroid 3D invasion assay ensued. The culmination of these protocols heralded the observation and imaging of clonal spheroids, approximately a decade after their initiation. Subsequent classification into invasive and noninvasive categories hinged upon the discernible presence or absence of cell protrusions 31. The methodologies harnessed to probe cellular dynamics and attributes, underpinning the pivotal steps of the investigation.

### Western blotting

Preparation and subsequent western blot analysis of protein lysates were meticulously conducted, in adherence to previously elucidated protocols 32. The primary antibodies enlisted for this purpose, along with their corresponding dilutions, were meticulously documented and furnished within Table Supplementary 1.

### RNA immunoprecipitation (RIP)

Employing the potent technique of RNA immunoprecipitation (RIP), an avenue for probing the physical interactions between RNA-binding proteins and RNA molecules, this study garnered insights into intricate molecular associations. For this purpose, a cohort of up to 10 million cells was dissociated and gathered, undergoing subsequent treatment with 10% formaldehyde and 2M glycine. Cell lysates, comprised of cell lysis buffer for western and immunoprecipitation (NCM Biotech, China), along with protease inhibitor (Bimake, China), phosphatase inhibitor (Bimake, China), and RNAse inhibitor (Accurate Biotechnology, China) were seamlessly amalgamated. Subsequent centrifugation at 1300g for 40 minutes at 4°C facilitated the collection of supernatant, which was then subjected to an 18-hour incubation with magnetic beads pre-treated with G3BP1 antibody (catalog: ab181150, Abcam, United Kingdom). As a negative control, normal rabbit IgG was judiciously employed. Post-incubation, the beads were meticulously cleansed with a carefully formulated cleaning buffer (constituting NaCl 100mM, Hepes 50mM, EDTA 5 mM, DTT 10 Mm, Triton X-100 0.5%, glycerinum 10%, and SDS 1%) over the course of an hour at 70°C. The ensuing supernatant was harnessed and extracted via Trizol (Accurate Biotechnology, China) 33.

### Quantitative RT-PCR (qRT-PCR) analysis

Isolation of total RNA from cells entailed the use of Trizol reagent, followed by reverse transcription of 1 μg of total RNA into first-strand cDNA utilizing a reverse transcriptase kit (Thermo Fisher, America). To quantify mRNA levels, qPCR was executed employing the SYBR® Green Premix Pro Taq HS qPCR Kit AG11701 (Accurate Biotechnology, China) on a Real-time fluorescence quantitative PCR system (Bio-Rad, USA). Glyceraldehyde-3-phosphate dehydrogenase (GAPDH) was enlisted as the internal control, with the 2-∆∆Ct method serving as the calculation methodology. Comprehensive insight into primer sequences employed for qRT-PCR, along with those pivotal for RIP, was collated within Table Supplementary 2 for qRT-PCR and Table Supplementary 3 for RIP.

### RNA pulldown assay

The RNA pulldown assay was conducted following a precedent study's protocol 34. The endeavor entailed the extraction of the biotin-coupled RNA complex, a process facilitated through the utilization of streptavidin magnetic beads (Beyotime, China). A pivotal element of this assay was the deployment of a 5' biotin-labeled oligonucleotide probe. Synthesized by RiboBio Technology (Guangzhou, China), this probe specifically targeted the junction site of JAK2, thereby honing in on a specific molecular region. The biotinylated JAK2 was subsequently captured employing streptavidin magnetic beads, and these beads, laden with biotinylated JAK2, were brought into contact with cell lysates. The overnight incubation at 4°C served as a milieu for the interactions to materialize. Post-incubation, meticulous washing and elution steps ensued, culminating in the analysis of proteins binding to the RNA complex through the conduit of western blotting.

### Fluorescence *in situ* hybridization (FISH) and immunofluorescence

Cultured cells were allotted a 24-hour growth period atop cover glass substrates. The initiation of the FISH assay unfolded with the application of a JAK2 mRNA FISH probe mix kit, thoughtfully crafted by RiboBio Technology (Guangzhou, China). Following the FISH experiment, the sequential undertaking of immunofluorescence ensued. A rabbit monoclonal antibody directed against G3BP1 (catalog: ab181150, Abcam, The United Kingdom) was entrusted with an overnight incubation period. Subsequent to this, the cells bore witness to staining with Horseradish Peroxidase goat anti-rabbit IgG-R (Dylight 488, Goat Anti-Rabbit IgG, catalog: #A23220, Abbkine). The cell nucleus was artfully stained with DAPI, imparting visual clarity to the ensuing images.

### Patient cohorts

The research encompassed a cohort of 324 cases involving paraffin-embedded specimens of NPC. Among these cases, 237 were males and 87 were females. These specimens were meticulously selected from the archives of the Department of Pathology at the Second Xiangya Hospital of Central South University, situated in Changsha, China. The span of patient follow-up ranged from January 2000 to December 2009, with certain cases under scrutiny for up to a decade. The NPC diagnosis was firmly established in accordance with the World Health Organization's histological classification for NPC, and staging was congruent with the parameters outlined in the 8th edition of the AJCC/UICC TNM staging system for NPC. Of note, none of the patients had undergone prior radiotherapy or chemotherapy at the juncture of the original biopsy. The research scope also encompassed the inclusion of 10 pairwise primary and recurrent specimens, 30 pairwise primary and metastasis specimens, as well as 53 instances of control nasopharyngeal epithelial tissues. The comprehensive portrayal of the patient cohort is meticulously cataloged within Table Supplementary 4, accompanied by the availability of comprehensive clinical records and follow-up data for all participants.

### Immunohistochemical staining and scoring

The Max Vision TM^+^ HRP-Polymer anti-Mouse IHC Kit was used to perform IHC staining for G3BP1, JAK2, and p-STAT3 proteins. Staining conditions for each antibody underwent calibration, drawing upon insights garnered from laboratory experience and in consonance with established protocols 8, 13, 35, 36. The primary antibody directed against G3BP1 was judiciously diluted at 1:300 (Monoclonal Mouse antibody, Catalog: sc-365338, SANTA CRUZ BIOTECHNOLOGY), while the primary antibody against JAK2 was employed at a dilution of 1:2000 (polyclonal Rabbit antibody, Catalog: A7694, ABclonal, China). The primary antibody against p-STAT3 witnessed a dilution of 1:250 (polyclonal Rabbit antibody, Catalog: YP0251, Immunoway, America). The experimental design incorporated positive control slides for each experiment, with matched IgG isotype antibody adopting the mantle of a negative control, assuring antibody specificity. Semi-quantitative evaluation, executed independently by two masked researchers, YZ and SF, constituted the bedrock for the assessment of staining intensity and extent for each slide. The intensity scores encompassed a scale of 0 (negative), 1 (weak), 2 (moderate), and 3 (strong), while staining extent scores traversed 0 (no staining), 1 (1-25%), 2 (26-50%), 3 (51-75%), and 4 (76-100%), dependent on the proportion of stained cells. The protein expression for each case was quantified by the product of the intensity score and tumor staining extent. Optimal cut-off levels, guided by published literature and practical considerations 8, 37, 38, provided the parameters for interpretation. Specifically, a staining index score of ≤ 6 signified negative expression for G3BP1, whereas ≥ 8 indicated positive expression. For JAK2, negative expression corresponded to a score of ≤ 1, while a score of ≥ 2 pointed toward positive expression. As for p-STAT3, a staining score of ≤ 4 indicated negative expression, while a score of ≥ 6 denoted positive expression. High-level concordance of 95% was achieved between the two evaluators, with any divergences resolved through discussion.

### Statistical analysis

The statistical analyses were performed using appropriate methods, which included the log-rank test, Chi-square test, multivariate Cox regression analysis, and Student's t-test with the assistance of SPSS for Windows (version 18.0, SPSS, Inc.) and GraphPad Prism (version 5.0, GraphPad Software Inc.) software packages. Statistical significance was set at *P* < 0.05. Standard deviation is shown by error bars in all figures. Noteworthy levels of statistical significance were indicated through the medium of asterisks, denoting degrees of differentiation: *P* < 0.05, *P* < 0.01, and *P* < 0.001, a stratification that aligned with the progressive strength of significance. Notably, the application of a two-tailed t-test was the bedrock for determining the statistical significance of the findings.

## Results

### Result 1: G3BP1 mRNA or protein was significantly higher expressed in multiple kinds of malignant tumors, including NPC

In this study, we searched for differential expression of G3BP1 mRNA in malignant tumors and their corresponding unpaired tissues (Figure Supplementary 1A) or paired tissues (Figure Supplementary 1B), including (Head and Neck Squamous Cell Carcinoma, HNSCC). We further explored the mRNA expression of G3BP1 in NPC via GEO database (GSE 12452), which suggested that G3BP1 mRNA expression was higher in NPC tissues compared with the adjacent tissues (Figure Supplementary 1C). Furthermore, the G3BP1 protein expression was detected in NPC cell lines and the immortalized nasopharyngeal epithelial cell line (NP69). As expected, compared with NP69, overexpression of G3BP1 was observed in NPC cell lines, especially in HNE2, 5-8F and HK1. In contrast, lower expression of G3BP1 (compared with NPC cell lines, rather than NP69) was founded in CNE1 and HONE1 (Figure supplementary 1D). Therefore, we chose HNE2, 5-8F and HK1 for the further knocking-down experiment, and HONE1 for overexpression experiment.

### Result 2: G3BP1 promoted proliferation and migration of NPC cells *in vitro*

**Figure 1:**
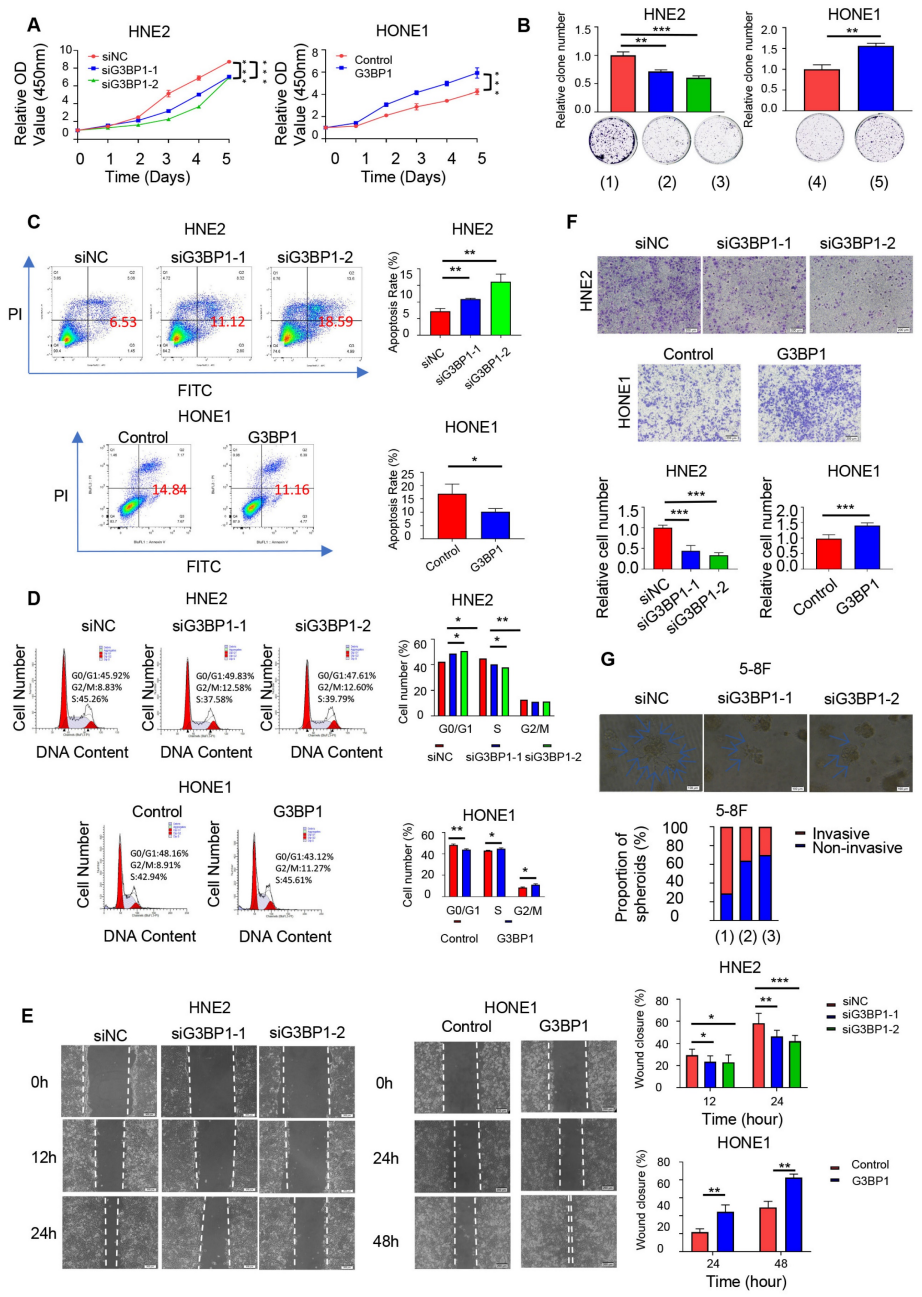
**G3BP1 was an oncogenetic factor in NPC cells.** (A and B) The effect of G3BP1 on cell proliferation was determined by CCK8 and clone formation in HNE2 and HONE1 cells respectively. Note: (1) siNC (control group), (2) siG3BP1-1, (3) siG3BP1-2, (4) Vector (control group), (5) Overexpression of G3BP1. (C) Knockdown of G3BP1 significantly increased the apoptosis rate, and overexpression of G3BP1 decreased the apoptosis rate in front of the starvation induction. (D) Cell cycle was arrested at G0/G1 phase under the condition of knocking-down of G3BP1 in HNE2 cells, and overexpression of G3BP1 promoted G0/G1 progression in HONE1 cells. (E) HNE2 cells with G3BP1 knock-down showed significantly lower migration rates, and HONE1 cells with G3BP1 overexpression showed significantly higher migration rates by wound healing assay. (F) HNE2 cells with G3BP1 knockdown showed significantly lower invasion rates in matrigel invasion assay compared with control cells. Overexpression of G3BP1 significantly increased invasion rates in matrigel invasion assay of HONE1 cells. (G) The number of spherical clones of invasive cells and prominent protrusions at the edges of the cells were significantly reduced with knocking-down of G3BP1 by three-dimensional invasion assay. Note: (1) siNC (control group), (2) siG3BP1-1, (3) siG3BP1-2.

We investigated the effects of G3BP1 on NPC cell lines and performed knockdown and overexpression experiments using two siRNAs and a G3BP1 plasmid, respectively. Knockdown of G3BP1 significantly inhibited cell growth in HNE2, 5-8F and HK1 cells, as shown by the CCK8 assay and colony formation assay (Figure 1A-B and Figure Supplementary 2A and 2B). Conversely, overexpression of G3BP1 promoted HONE1 cell growth (Figure 1A-B). Furthermore, knockdown of G3BP1 increased the apoptosis rate, while overexpression of G3BP1 decreased the apoptosis rate in front of starvation induction (Figure 1C and Figure Supplementary 2C). In addition, knockdown of G3BP1 arrested the cell cycle at G0/G1 phase, while overexpression of G3BP1 promoted G0/G1 progression (Figure 1D). Notably, in 5-8F cells, the cell cycle was arrested at G2/M phase (Figure Supplementary 2D). Furthermore, we observed that knockdown of G3BP1 significantly reduced cell migration and invasion rates in wound healing and matrigel invasion assays (Figure 1E-F and Figure Supplementary 3A and 3B). The cell mobility and invasion ability were also detected by a three-dimensional invasion assay, and the number of spherical clones of invasive cells and prominent protrusions at the edges of the cells were significantly reduced with G3BP1 knockdown (Figure 1G).

### Result 3: G3BP1 orchestrated cell proliferation, migration, and invasion via the JAK2/STAT3 signaling pathway

**Figure 2:**
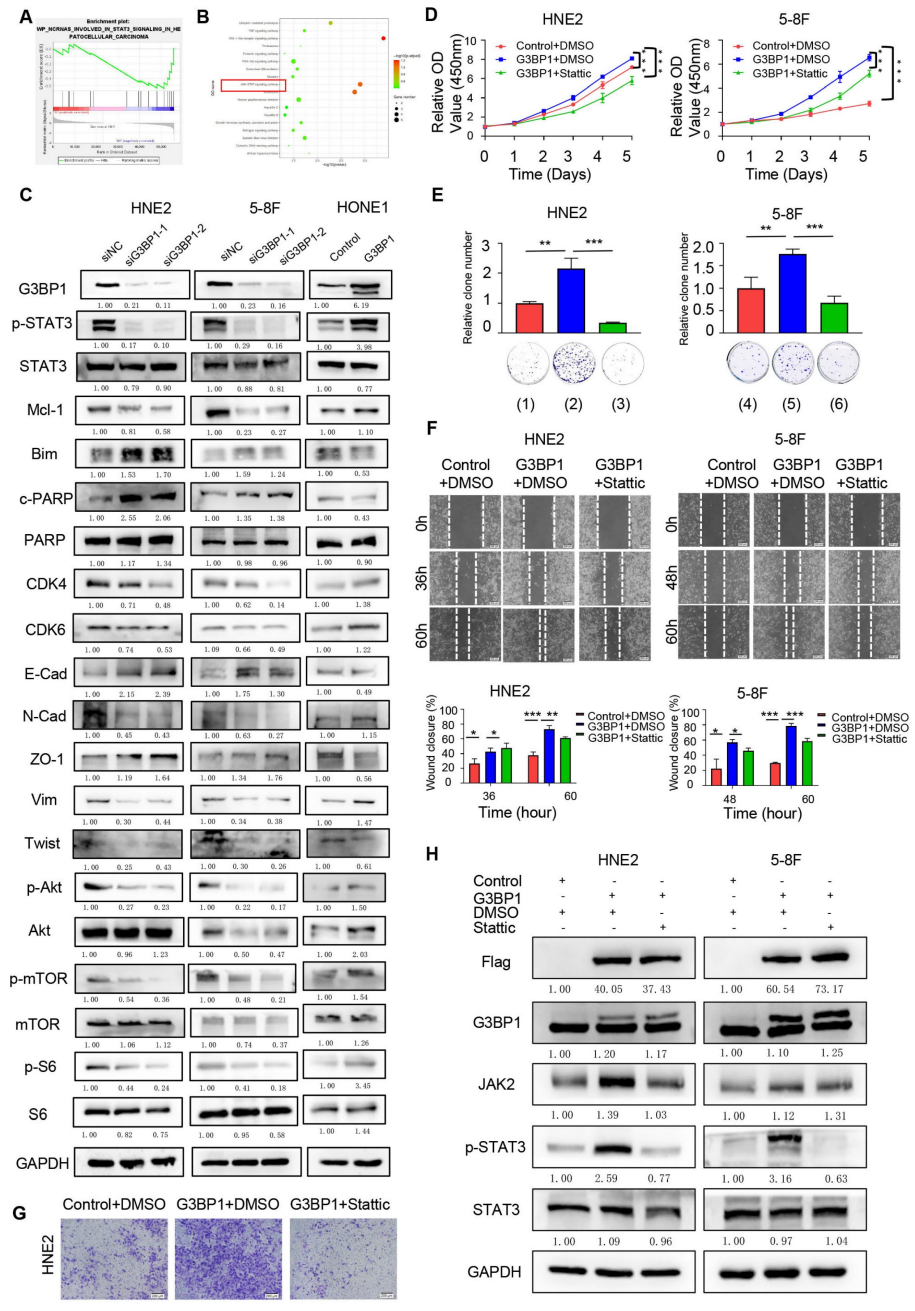
**G3BP1 promoted cell proliferation, migration and invasion by up-regulating phosphoration of STAT3.** (A and B) The differential expression genes were identified in HNE2 and HONE1 cells after knockdown or overexpression of G3BP1, and potential molecular mechanisms were suspected by GSEA and KEGG analysis. (C) G3BP1 could regulate the expression of p-STAT3 positively, and the downstream proteins of STAT3 signaling were also regulated. The Akt/mTOR signaling was also regulated by G3BP1. (D and E) Stattic was used to recover the cell growth of HNE2 and 5-8F with overexpression of G3BP1 (CCK8 assay and clone formation). Note: (1) Control+DMSO, (2) G3BP1+DMSO, (3) G3BP1+Stattic, (4) Control+DMSO, (5) G3BP1+DMSO, (6) G3BP1+Stattic. (F) Stattic might reverse the migration rates in wound healing assay of HNE2 cells with overexpression of G3BP1. (G) Stattic might recover the invasion rates in matrigel invasion assay of HNE2 and 5-8F with overexpression of G3BP1. (H) p-STAT3 was up-regulated by overexpression of G3BP1, which could be recovered by stattic.

To investigate the potential mechanism of G3BP1 in NPC cells, we performed RNA-seq to detect differentially expressed genes following knockdown or overexpression of G3BP1 in HNE2 and HONE1 cell lines. We then used GSEA and KEGG pathway analysis to examine the potential signaling pathways affected by G3BP1. Our findings suggested that several signaling pathways were related to G3BP1, including PI3K/Akt and JAK/STAT signaling pathways (Figure 2A-B). Further, we evaluated the expression of phosphorylated STAT3, total STAT3, and its downstream targets via western blotting. Our results showed that knockdown of G3BP1 resulted in downregulation of phosphorylated STAT3, while overexpression of G3BP1 induced upregulation of phosphorylated STAT3, without significant changes in total STAT3 expression. To gain deeper insights into the downstream targets of JAK/STAT3 signaling, we examined Mcl-1, Bim, and cleaved PARP. Our observations showed that knockdown of G3BP1 in HNE2, 5-8F and HK1 cells resulted in downregulation of Mcl-1 and upregulation of Bim and cleaved PARP. Overexpression of G3BP1, on the other hand, had the opposite effect. Further, we examined CDK4 and CDK6, which showed the homodromous regulation of G3BP1. We also investigated the expression of proteins involved in the epithelial-mesenchymal transition (EMT) process and found that N-Cadherin, Vimentin, and twist protein were downregulated, while the expression of E-Cadherin and ZO-1 protein was upregulated following knockdown of G3BP1. Similar results were obtained in HONE1 cells with overexpression of G3BP1, supporting the preliminary regulation relationship between G3BP1 and JAK/STAT3 signaling (Figure 2C and Figure Supplementary 4A). Moreover, it was also detected the expression of PI3K/Akt signaling, and decreased expression of p-Akt, p-mTOR and p-S6 was observed in cells with knockdown of G3BP1, not accompanying the change of total protein. Correspondingly, overexpression of G3BP1 might contribute the activation of PI3K/Akt signaling (Figure 2C and Figure Supplementary 4B). The regulation of G3BP1 and PI3K/Akt signaling was proved in several studies, and our results also certificated it 8, 10. The verification of PI3K/Akt signaling supported the accuracy of the RNA-seq, and it was selected JAK/STAT3 signaling pathway for the following research in consideration of innovation.

To confirm the role of JAK/STAT3 signaling in mediating the effects of G3BP1, we applied stattic, a non-peptidic small molecule that selectively inhibits dimerization and nuclear translocation of STAT3, to neutralize JAK/STAT3 signaling activation. Our observations showed that stattic rescued the phenotypes caused by G3BP1 overexpression, including cell proliferation (CCK8 assay and colony formation assay, Figure 2D-E and Figure Supplementary 5A and 5B), migration (wound healing assay, Figure 2F and Figure Supplementary 5C), and invasion (matrigel invasion assay, Figure 2G and Figure Supplementary 5D), as well as the activation of JAK/STAT3 signaling (phosphorylated STAT3), without affecting the expression of total STAT3 (Figure 2H and Figure Supplementary 5E).

### Result 4: G3BP1 was an RNA binding protein and interacted with JAK2 mRNA

**Figure 3:**
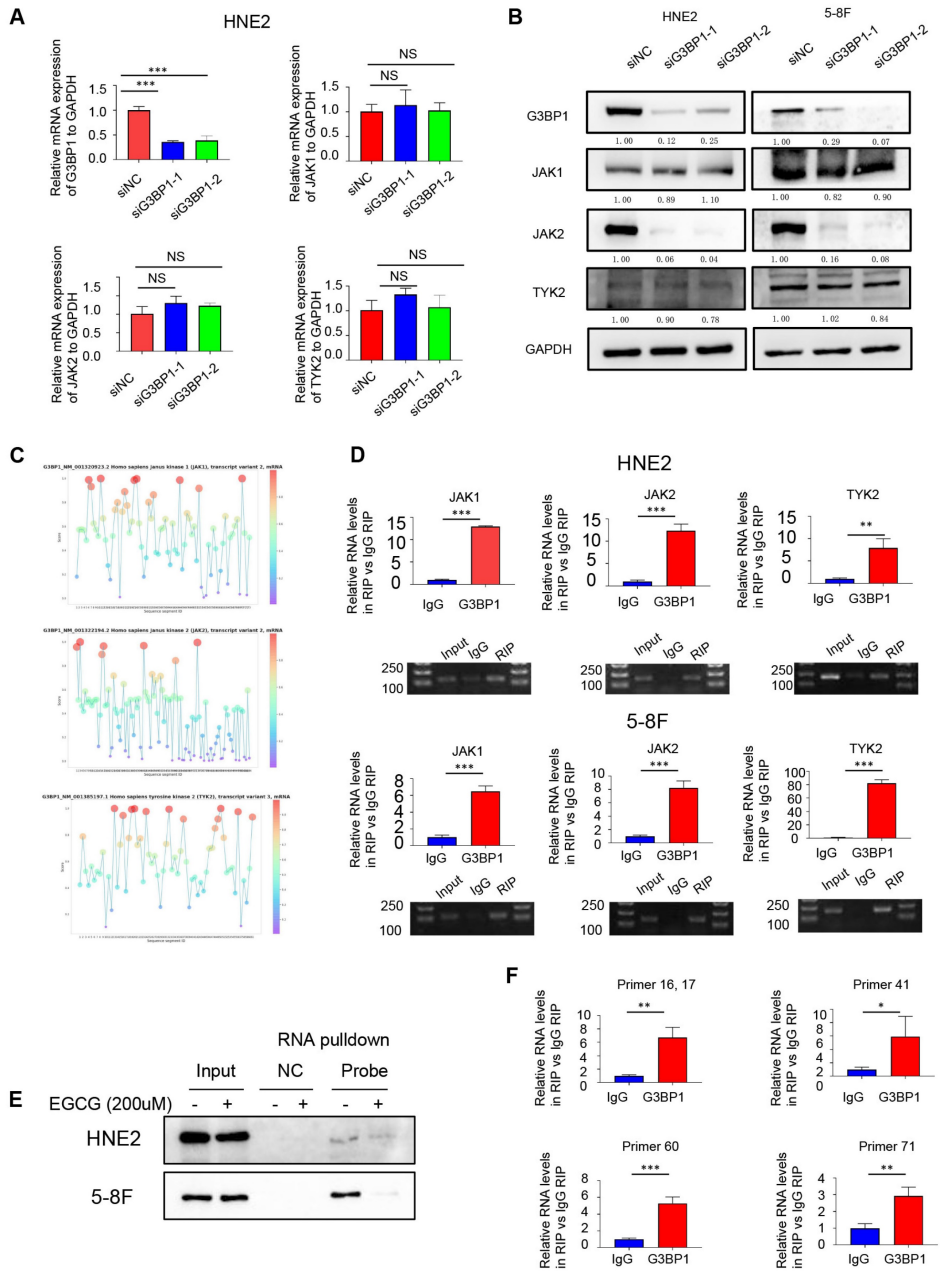
** G3BP1 was an RNA binding protein to interact with JAK2 mRNA.** (A) G3BP1 didn't influence the mRNA expression of JAK1, JAK2 and TYK2. (B) Knocking down G3BP1 could downregulated protein expression of JAK2, but not JAK1 and TYK2. (C) G3BP1 was potential to interact with three JAKs mRNA via RBPsuite database. (D) The binding of G3BP1 protein and JAK2 mRNA was validated by RNA binding protein immunoprecipitation assay (RIP assay). (E) JAK2 mRNA could bind G3BP1 protein by RNA pulldown assay, and the interaction could be broken by EGCG. (F) The potential binding sites of G3BP1 protein and JAK2 mRNA were preliminarily verified by ultrasonic interruption of RNA and the structural inhibitor of G3BP1.

Based on the previous study, it was supposed to investigate the specific JAKs and the regulatory mechanism through which G3BP1 affects the JAK/STAT3 signaling pathway. There was no significant change of JAKs mRNA expression when knocking-down or overexpression of G3BP1 (Figure 3A). Interestingly, the expression of JAK2 protein was reduced followed by knocking-down of G3BP1, while there was no change of JAK1 and TYK2 expression (Figure 3B).

Next, we explored whether G3BP1 played an oncogenic role via its RNA binding ability. To predict potential RNA-protein interactions, we used the RNAct website, which predicted JAK2 with a relatively high score, and JAK1 was verified with eCLIP data (Figure Supplementary 6A). We also utilized RBPsuite to predict the possible binding sites of G3BP1 and the JAKs family members, and surprisingly, all three JAKs were predicted to bind with G3BP1 protein, with several possible binding sites suggested (Figure 3C).

To verify the combination of protein and RNA, we performed an RNA immunoprecipitation (RIP) assay. We conjugated G3BP1 protein to magnetic beads coated with G3BP1 antibody, extracted the binding RNA, and performed reverse transcription and quantitative polymerase chain reaction (qPCR) using primers designed for JAK1, JAK2, and TYK2. We then verified the qPCR products through DNA gel electrophoresis and calculated the fold change of RIP group and control group (IgG group) by exporting the qPCR data (Figure 3D). The protein that was pulled down by JAK2 mRNA was then detected using a G3BP1 antibody through western blotting. The results of this experiment indicated that G3BP1 protein and JAK2 mRNA do indeed bind to each other (Figure Supplementary 6B).

Epigallocatechin gallate (EGCG), a potent antioxidant that is isolated from green tea, has been reported as a structural inhibitor of G3BP1 by occupying the RGG motif and RAS-GAP binding region (amino acids 225-340) and has the potential to decrease cGAS activation 40. It was also conducted molecular docking analysis for the G3BP1 NTF2 domain, and the results showed that the binding energy of EGCG and G3BP1 NTF2 domain is -9.67 kcal/mol, suggesting stable binding via Autodock Vina V1.2.2 (Figure Supplementary 6C). Interestingly, the tight interaction of G3BP1 and JAK2 mRNA was broken by EGCG, which hinted the possible binding domain of G3BP1 in interacting with JAK2 mRNA (Figure 3E).

To predict the potential binding sites between G3BP1 and JAK2 mRNA, we used the RBPsuite website to cut the JAK2 mRNA sequence into 101 segments and calculate the scores for each segment (Figure Supplementary 6D). We then designed several primers to verify the prediction, and observed significant differences in the fold change of the RIP group (primers 16, 17, 41, 60, and 71) compared to the control group (IgG group), providing clues about the specific binding site of G3BP1 and JAK2 mRNA (Figure 3F).

We also confirmed that the binding of G3BP1 and JAK2 mRNA occurs independently of SGs formation. Immunofluorescence and FISH assays were used to detect the location of G3BP1 and JAK2 mRNA, respectively. We observed extensive distribution of JAK2 mRNA in the cytoplasm and nucleus, while G3BP1 was mainly found in the cytoplasm. Interestingly, G3BP1 assembled into light spots without any stimulation, whereas JAK2 mRNA did not, suggesting that the binding of G3BP1 and JAK2 mRNA is not dependent on SGs formation (Figure Supplementary 6E).

### Result 5: JAK2 promoted proliferation and migration *in vitro*

In our quest to decode the intricate correlation between JAK2 and G3BP1, we ventured into the unexplored field of JAK2's role in NPC. With meticulous precision, we conducted an array of experiments to illuminate the oncogenic roles that JAK2 could play. We used CCK8 and clone formation assay to confirm the oncogenic roles of JAK2 in cell proliferation. Knockdown of JAK2 was found to decrease the proliferation of HNE2 and 5-8F cells (Figure Supplementary 7A and 7B). In addition, wound healing assay and matrigel invasion assay were used to verify that knockdown of JAK2 reduced migration and invasion rates (Figure Supplementary 7C, 7D and 7E).

### Result 6: JAK2 reversed the effect of G3BP1 in NPC cells

**Figure 4:**
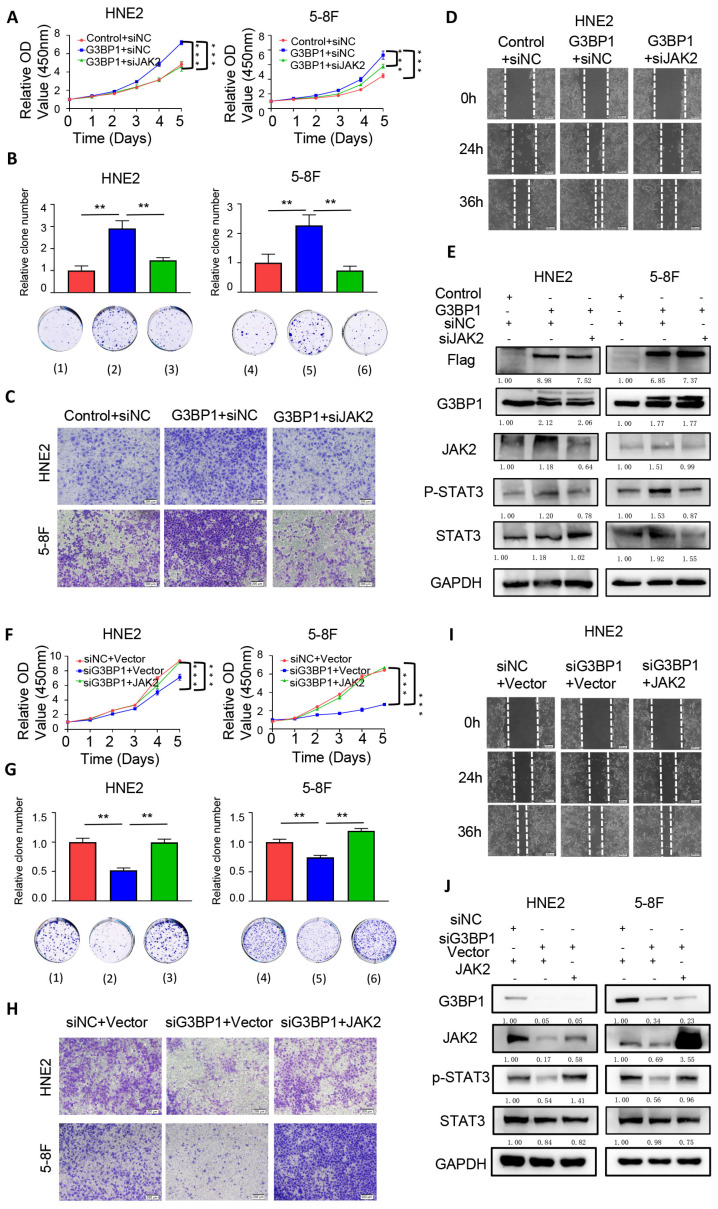
** JAK2 could reverse the effect of G3BP1 in NPC cells.** (A and B) knocking down JAK2 might reverse the cell growth of HNE2 and 5-8F with overexpression of G3BP1 by CCK8 assay and clone formation. Note: (1) Control+siNC, (2) G3BP1+siNC, (3) G3BP1+siJAK2, (4) Control+siNC, (5) G3BP1+siNC, (6) G3BP1+siJAK2. (C) Knocking down JAK2 might reverse the invasion rates in matrigel invasion assay of HNE2 and 5-8F with overexpression of G3BP1. (D) Knocking down JAK2 might reverse the migration rates in wound healing assay of HNE2 with overexpression of G3BP1. (E) p-STAT3 was up-regulated by overexpression of G3BP1, which could be recovered by knock-down of JAK2. (F and G) Overexpression of JAK2 might reverse the cell growth of HNE2 and 5-8F with knocking down G3BP1 by CCK8 assay and clone formation. Note: (1) siNC+Vector, (2) siG3BP1+Vector, (3) siG3BP1+JAK2. (4) SiNC+Vector, (5) siG3BP1+Vector, (6) siG3BP1+JAK2. (H) Overexpression of JAK2 might reverse the invasion rates in matrigel invasion assay of HNE2 and 5-8F with knocking down G3BP1. (I) Overexpression of JAK2 might reverse the migration rates in wound healing assay with knocking down G3BP1 in HNE2 cells. (J) Knocking down G3BP1 could decrease the expression of p-STAT3, which might be recovered by overexpression of JAK2.

The previous findings suggested that G3BP1 interacts with JAK2 mRNA and regulates JAK2 protein expression, thereby activating the JAK2/STAT3 signaling pathway. We sought to investigate whether the overexpression or knockdown of JAK2 could rescue or impair the function of G3BP1 in JAK2/STAT3 signaling activation. As expected, knocking down JAK2 rescued the malignant phenotype resulting from G3BP1 overexpression, while overexpression of JAK2 partially rescued the impaired malignant phenotype induced by G3BP1 knockdown (Figure 4A-4D, Figure 4F-4I, Figure Supplementary 8 and 9). In addition to these phenotypes, we also detected the expression of phosphorylated STAT3 and total STAT3 in the same groups mentioned above. We observed that the expression of phosphorylated STAT3 was reduced upon G3BP1 knockdown, while JAK2 overexpression partially rescued the roles of G3BP1 (Figure 4J). Correspondingly, knocking down JAK2 counteracted the activation of the JAK2/STAT3 pathway by G3BP1 (Figure 4E).

### Result 7: G3BP1 promoted proliferation and migration *in vivo*

**Figure 5:**
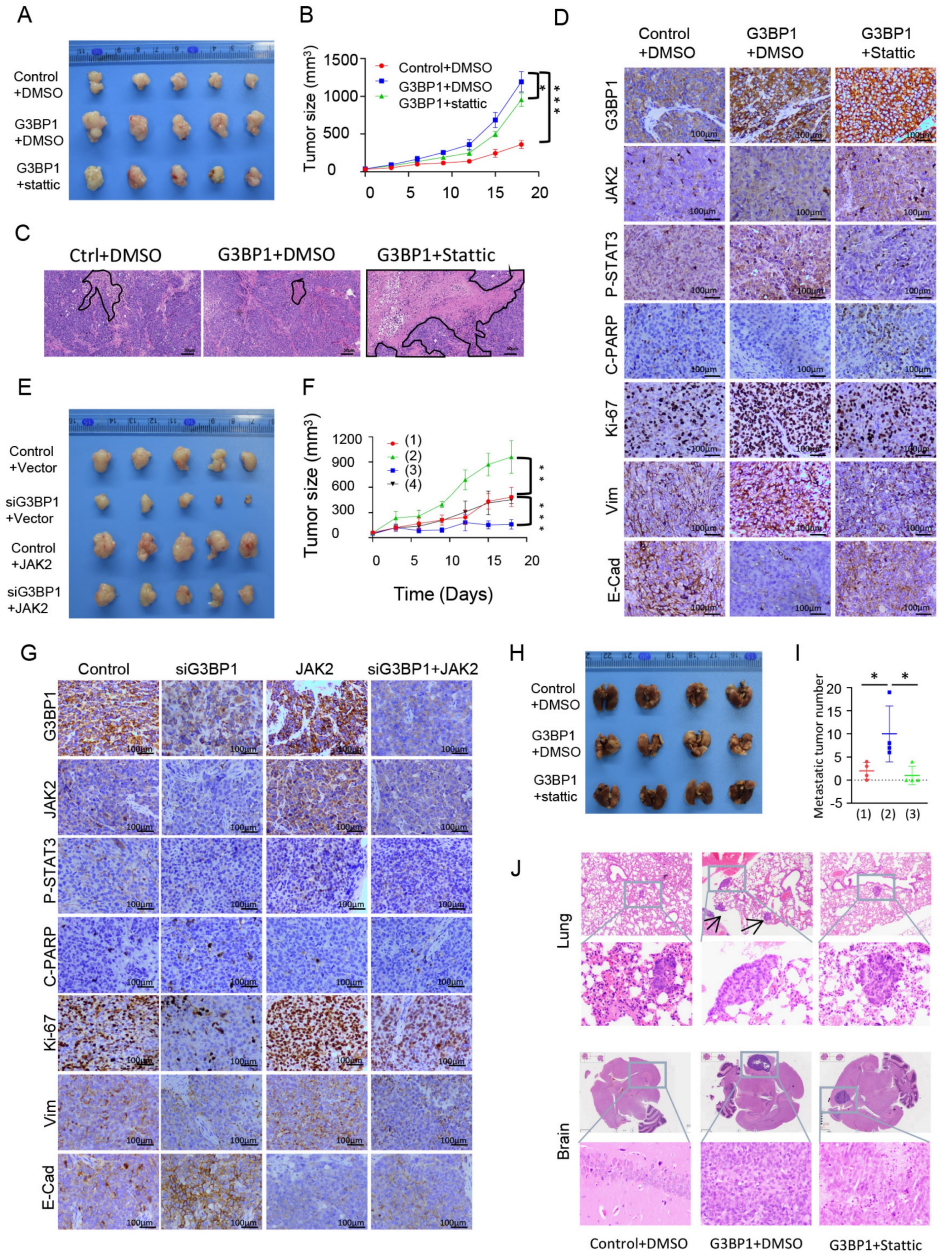
** G3BP1 promoted cell proliferation and increased metastasis *in vivo*.** (A and B) The roles of G3BP1 on cell growth *in vivo* were verified by tumorigenesis assay in nude mice. (C) Representative image of H&E staining of xenograft model, and the necrosis area were drawn. (D) Representative image of IHC staining of xenograft model. (E and F) Mice model was constructed to verify the oncogenic roles of G3BP1 and JAK2, moreover, JAK2 could rescue the slackening growth induced by knocking-down of G3BP1. Note: (1) siNC+Vector, (2) siG3BP1+Vector, (3) siNC+JAK2, (4) siG3BP1+ JAK2. (G) Representative image of IHC staining of xenograft model. (H) The effects of G3BP1 on the metastasis *in vivo* were confirmed by tail vein metastasis model, and stattic was injected for rescue assay. (I) The number of metastatic nudes in lungs under microscope. Note: (1) Control+DMSO, (2) G3BP1+DMSO, (3) G3BP1+Stattic. (J) Representative image of H&E staining of metastatic nudes, and arrows indicated metastatic tumors.

To further verify the roles of G3BP1 in cell proliferation, as well as the therapeutic and rescue functions of stattic, subcutaneous tumorigenesis in nude mice was conducted. The volume and weight of tumors were significantly increased in the group injected with stable overexpression of G3BP1 cells, and stattic partially rescued the oncogenic roles of G3BP1, decreasing the volume and weight of tumors (Figure 5A, Figure Supplementary 10A and Figure Supplementary 10B). The relative tumor growth curves were shown in Figure 5B. The G3BP1-injected group showed significantly increased tumor growth compared to the control group, while the stattic-treated group reduced tumor growth compared to the G3BP1 overexpression group. It is worth noting that stattic significantly increased the area of necrosis compared to the other two groups in H&E staining slides (Figure 5C).

In agreement with our *in vitro* studies, the expression of phosphorylated STAT3 in xenografts was significantly increased in the G3BP1 group and reduced in the stattic group. The expression of JAK2, cleaved PARP, Ki-67, Vim, and E-Cadherin were detected, and the tendency was as expected (Figure 5D and Figure Supplementary 10D). The tissues were mashed, extracted, and detected by western blotting, and G3BP1 increased the expression of phosphorylated STAT3, while stattic rescued the activation of the signaling (Figure Supplementary 10C).

To further validate the oncogenic roles of G3BP1 and JAK2, and the potential rescue effect of JAK2 on the growth inhibition caused by G3BP1 knockdown, we established another mouse model. We measured the volume and weight of tumors, which showed consistent results with the growth curve analysis (Figure 5E, Figure 5F, Figure Supplementary 11A and Figure Supplementary 11B). We found that the expression of JAK2, phosphorylated STAT3, Ki-67, and Vimentin decreased, while cleaved PARP and E-Cadherin increased upon knocking-down of G3BP1. Overexpression of JAK2 had the opposite effect, and JAK2 could partially rescue the effects of G3BP1 knockdown (Figure 5G and Figure Supplementary 11C).

To establish a metastatic tumor model, we injected 5-8F cells via tail vein. The experimental group was consistent with subcutaneous transplantation tumor. We observed and quantified the number of metastatic tumors in the lung and brain of mice. Consistent with the subcutaneous tumorigenesis model, we found that the number of lung metastatic tumors was significantly higher in the G3BP1 overexpression group than in the control group, and injection of stattic partially reduced the number of tumors (Figure 5H-5J). In the brain metastasis model, we observed that the tumor size was larger in the G3BP1 overexpression group than in the stattic injection group, and no clear tumor was found in the control group (Figure 5J and Figure Supplementary 11D). To confirm the results, we performed IHC staining for CK8/18, a classical marker for squamous cells (Figure Supplementary 11E).

### Result 8: Expression of G3BP1, JAK2 and p-STAT3 proteins associated with clinical progression of NPC

**Figure 6:**
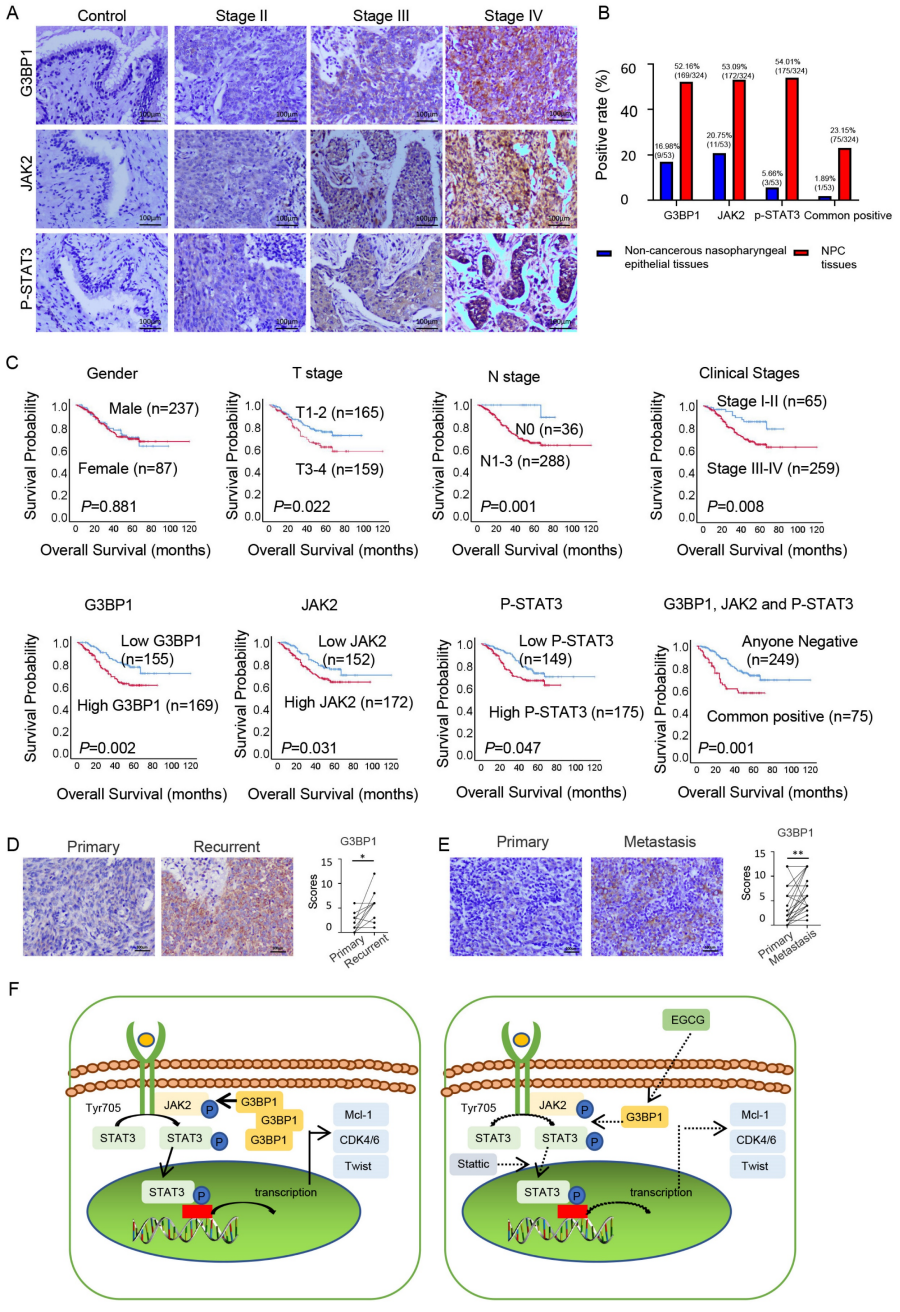
** Expression of G3BP1, JAK2 and p-STAT3 predicted overall survival of patients with NPC.** (A) Weak positive expression of G3BP1, JAK2 and p-STAT3 were indicated in the columnar epithelial cells of non-cancerous nasopharyngeal tissue; stronger positive expression of G3BP1, JAK2 and p-STAT3 were indicated in NPC tissues with the enhanced clinical stages. (B) The statistical analysis of positive rates of expression of G3BP1, JAK2 and p-STAT3 independently and commonly. (C) The overall survival rates were significantly lower for NPC patients with positive expression of G3BP1, JAK2 and p-STAT3 independently and commonly, as well as higher T stages, N stages and clinical stages. (D) Representative image and statistical analysis of IHC staining of G3BP1 in pairwise primary and recurrent tumors. (E) Representative image and statistical analysis of IHC staining of G3BP1 in pairwise primary and metastasis tumors. (F) Schematic representation of molecular mechanism of G3BP1 in promoting tumor growth and metastasis.

In the realm of clinical investigations, we aimed to confirm whether G3BP1, JAK2, and p-STAT3's roles unfurled as pivotal players in the NPC progression. We conducted IHC to detect the expression and cellular location of G3BP1, JAK2, and p-STAT3 in NPC and non-cancerous nasopharyngeal epithelial tissues. Our findings revealed that cancer cells had diffuse staining for G3BP1 in the membrane and cytoplasm, but nuclear staining was rarely observed. Cytoplasm staining was seen for JAK2, while p-STAT3 displayed two basic models, namely cytoplasm staining-oriented and nuclear staining-oriented. NPC tissues had higher expression of G3BP1, JAK2, and p-STAT3 compared to control tissues, with higher expression levels observed in advanced stages (Figure 6A and 6B). We also examined the associations between the expression of G3BP1, JAK2, p-STAT3, and all three proteins and clinicopathological features of NPC, including gender, age, T stage, lymph node status (N stage), M stage, and clinical stages. Univariate Chi Square Test results indicated that the expression of G3BP1 had a positive relationship with advanced T stage, N stage, and clinical stages, while the expression of JAK2 had a positive relationship with N stage. Additionally, the expression of p-STAT3 had a positive relationship with N stage and clinical stages of NPC patients. We also found that co-expression of G3BP1, JAK2, and p-STAT3 was associated with N stage and clinical stages of NPC patients (Table 1). Pairwise clinical specimens were analyzed for G3BP1, and we observed that recurrent tumors had significantly higher expression than primary tumors, as well as metastases compared to primary tumors (Figure 6D and Figure 6E).

Univariate survival analysis (log-rank test) showed that the OS rates was significantly lower for NPC patients with positive expression of G3BP1, JAK2 and p-STAT3 independently or commonly (Figure 6C). In multivariate analysis of the features of patients with NPC, positive expression of G3BP1 is identified as an independent poorer prognostic factor for patients with NPC, along with lymph node metastasis and distant metastasis (Table 2). These findings highlight the potential of G3BP1, JAK2, and p-STAT3 as biomarkers for NPC prognosis and therapy.

## Discussion

G3BP1 is a multi‐functional protein that is well known for its roles in involvement in stress granule (SG) assembly and dynamics. SG formation confers survival advantages and chemotherapeutic resistance to cells. The formation and assembly of SGs have been implicated in the occurrence and progression of many tumor-related or unrelated diseases 7, 41, 42. In our previous exploration 13, YB1's interplay with G3BP1 in NPC cells drew attention, forming SGs in response to arsenic trioxide stimulation. However, the roles that G3BP1 assumes beyond the realm of SGs in NPC cells remained enigmatic.

In fact, G3BP1 has been reported to play oncogenic function independent of SGs formation in some malignant tumors. In our previous study, we demonstrated that the G3BP1mRNA and protein expression was higher in NSCLC tissues, and multivariate analysis confirmed that overexpression of G3BP1 protein was an independent poorer prognostic factor for NSCLC patients 8. Additionally, knockdown of G3BPs was found to suppress the growth, migration and invasion capability of human lung carcinoma H1299 cells by inhibiting the phosphorylation of Src, FAK, ERK and the levels of NF-κB 43. G3BP1 was also shown to bind lncRNA P53RRA, and displaced p53 from G3BP1 complex, and result in greater p53 retention in the nucleus, leading to cell-cycle arrest, apoptosis, and ferroptosis 44. Moreover, G3BP1 was also considered to inhibit proliferation of NSCLC cells by controlling cellular senescence via activating the NF-κB and STAT3 pathways through cyclic GMP-AMP synthase (cGAS) 45. Besides NSCLC, G3BP1 was reported to participate in proliferation of breast cancer by coordinating with GSK-3β and stabilizing β-catenin 11, and peripheral myelin protein 22 (PMP22) was also considered to be involved in 46. In other malignant tumors, G3BP1 was thought to play the oncogenic roles such as gastric cancer 12, 47-49, prostate cancer 50, 51, esophageal cancer 10, ovarian cancer 52, 53 and colorectal cancer 54. It has been reported that G3BP1 could inhibit cell proliferation, and low G3BP1 levels enhanced mTORC1-driven breast cancer cell motility and correlate with adverse outcomes in patients 55. In our study, univariate and multivariate analyses showed that G3BP1 expression was an independent poorer prognostic factor for NPC patients, indicating that G3BP1 may play a similar oncogenic role in NPC as in other malignancies. These findings suggest that G3BP1 could be a potential therapeutic target for the treatment of NPC.

As a classical RNA binding protein, G3BP1 was known to bind both coding and non-coding RNAs and mRNAs through its three conserved domains: nuclear transporter factor 2 (NTF2) domain, RNA-recognition module (RRM) and RGG (arginine-glycine-glycine) motif 40, 56. The NTF2-like domain is thought to be involved in G3BP dimerization, SGs assembly, and binding to various proliferation-related proteins such as Ras GTPase Activating Protein (rasGAP) 57. RRM and RGG domains have been shown to be important for the localization of G3BP1 to virus factories (VFs), where viral transcription, translation, and replication occur during mammalian orthoreovirus (MRV) infection 58. Additionally, RGG domain of G3BP1 can bind directly to RNA guanine quadruplexes (rG4) structures, and RRM domain enhanced the selective binding 59.

Through its RNA-binding ability, G3BP1 has been implicated in regulating various malignant phenotypes by binding to both coding and non-coding RNAs, including lncRNAs and mRNAs 44, 60, 61. G3BP1 has been shown to bind to m5C-modified H19 lncRNA, leading to MYC accumulation 60. It also binds to other lncRNAs, such as lncRNA SPOCD1-AS and lncRNA P53RRA 44, 60. G3BP1 can bind to mRNA and guide selective translation and stress adaptation in cancer 61. It has also been shown to regulate intra-axonal mRNA translation, affecting axon growth in cultured neurons and stress granule-like structures in axons. Moreover, G3BP1 facilitates the interaction of G3BP1 to E2F1 3'-untranslated region, thereby stabilizing E2F1 mRNA 62. Interestingly, the natural compound EGCG has been shown to inhibit G3BP1 through its binding to the RGG motif and the Ras-GAP binding region (amino acids 225-340) 40, 63. It remains to be investigated whether EGCG can disrupt the interaction of G3BP1 and its bound RNAs.

The JAKs/STAT3 signaling pathway is a well-established oncogenic pathway that regulates a range of cellular processes including cell proliferation, differentiation, apoptosis, angiogenesis, immunosuppression, and sustained inflammation 14, 15. Various factors are known to influence JAKs/STAT3 activation, including RNA binding proteins. For instance, DENR, an RNA binding protein, was shown to counteract the translational repression of three consecutive upstream open reading frames (uORFs) upstream of JAK2, thus regulating JAK2 translation and the IFNγ/JAK/STAT signaling pathway, resulting in reduced PD-L1 expression in tumors 23. Additionally, NONO, another RNA binding protein, is found to bind to STAT3 mRNA, increasing its levels in triple-negative breast cancer. Furthermore, NONO directly interacted with STAT3 protein, increasing its stability and transcriptional activity, thus contributing to oncogenic function 64. Another RNA binding protein, AT-rich interactive domain-containing protein 5a (Arid5a) has also been shown to regulate autoimmunity by stabilizing interleukin-6 and STAT3 mRNAs to act on the JAKs/STAT3 signaling pathway 65, 66. Despite the evidence suggesting that G3BP1 might influence JAKs/STAT3 signaling pathway activation, with previous studies indicating that G3BP1 can activate NF-κB and STAT3 pathways through cGAS, and further promote senescent-associated secretory phenotype senescent-associated secretory phenotype 45, it remains unclear how G3BP1 regulates STAT3 signaling as an RNA binding protein, and the potential binding sites require further elucidation. Notably, G3BP1 has been shown to promote tumor progression and metastasis through the IL-6/G3BP1/STAT3 signaling axis in renal cell carcinoma 67. However, these studies mentioned above don't illustrate how G3BP1 regulate STAT3 signaling as a RNA binding protein, and the potential binding sites.

## Conclusion

This paper rooted in comprehensive analyses of G3BP1 mRNA and protein expression, the study unveils the oncogenic prowess of G3BP1, its orchestration of the JAK2/STAT3 signaling pathway, and its pivotal role in NPC progression. Elucidating the molecular mechanism, the study undertook an array of experiments, unearthing G3BP1's multifaceted role in NPC cell proliferation, migration, and its intimate engagement with the JAK2/STAT3 signaling pathway. Notably, the binding of G3BP1 to JAK2 mRNA emerged as a crucial juncture, one disrupted by the potent antioxidant EGCG, resonating with its therapeutic potential. Intriguingly, G3BP1's influence on the JAK2/STAT3 pathway was unveiled as a separate entity, distinct from the formation of stress granules (SGs), unraveling an independent narrative of regulation. The study's orchestration of rescue experiments further validated the intricate choreography between G3BP1, JAK2, and STAT3, culminating in the revelation of G3BP1's promotion of proliferation and migration through *in vivo* tumorigenesis and metastatic models. The exploration culminated with investigating the clinical realm, where G3BP1's prominence in NPC tissues was illuminated through IHC analysis. Moreover, the study established G3BP1, JAK2, and p-STAT3 as powerful prognostic markers, with G3BP1's expression standing as an independent indicator of poorer outcomes for NPC patients.

However, even as this study sheds light on G3BP1's intricate roles in NPC, it acknowledges the limitations that temper its findings. A spotlight is cast on the future, where SGs-related functions of G3BP1 beckon exploration, offering a deeper understanding of its role in NPC initiation and progression. The investigation forward also includes delving into G3BP1's therapeutic potential for NPC, and perhaps beyond.

## Supplementary Material

Supplementary figures and tables.

## Figures and Tables

**Table 1:** Association between expression of G3BP1, JAK2 and P-STAT3 proteins and clinicopathological features of NPC

Clinicopathological features	G3BP1			JAK2			P-STAT3			G3BP1, JAK2 and P-STAT3		
	N (%)	P (%)	*P-Value*	N (%)	P (%)	*P-Value*	N (%)	P (%)	*P-Value*	N (%)	P (%)	*P-Value*
**Gender**			0.177			0.963			0.998			0.351
Male (n=237)	108	129		111	126		109	128		179	58	
Female (n=87)	47	40		41	46		40	47		70	17	
**Age**			0.711			0.213			0.415			0.269
<40 (n=57)	26	31		31	26		29	28		47	10	
≥40 (n=267)	129	138		121	146		120	147		202	65	
**T stage**			0.004**			0.754			0.487			0.103
T1 and T2 (n=165)	92	73		76	89		79	86		133	32	
T3and T4 (n=159)	63	96		76	83		70	89		116	43	
**N stage**			0.041*			0.030*			0.008**			0.025*
N0 (n=36)	23	13		23	13		24	12		33	3	
N1/N2/N3(n=288)	132	156		129	159		125	163		216	72	
**M stage**			0.096			0.636			0.470			0.340
M0 (n=321)	155	166		151	170		147	174		246	75	
M1(n=3)	0	3		1	2		2	1		3	0	
**Clinical stages**			0.013*			0.210			0.048*			0.003**
Ⅰ and Ⅱ (n=65)	40	25		35	30		37	28		59	6	
III and Ⅳ (n=259)	115	144		117	142		112	147		190	69	

*: χ^2^, *P* <0.05 (2-tailed); **: χ^2^, *P* <0.01 (2-tailed

**Table 2:** Summary of multivariate of Cox proportional regression for overall survival in 324 cases of NPC

Parameter	SE	Wald	Sig.	Exp (B)	95.0% CI for Exp (B)	
					Lower	Upper
**Gender**	0.258	0.023	0.880	0.962	0.580	1.596
**Age**	0.333	0.086	0.769	0.907	0.472	1.741
**T stages**	0.251	1.883	0.170	0.709	0.434	1.159
**N stages**	1.016	5.123	0.024*	0.100	0.014	0.735
**M stages**	0.639	8.385	0.004**	0.157	0.045	0.550
**Clinical stages**	0.392	0.598	0.439	0.739	0.343	1.592
**G3BP1**	0.243	3.898	0.048*	0.619	0.384	0.997
**JAK2**	0.238	1.427	0.232	0.753	0.472	1.200
**P-STAT3**	0.241	1.067	0.302	0.779	0.486	1.251

Abbreviations: CI, confidence interval; Exp (β), odds ratio. Note: multivariate analysis of Cox regression, ^*^P<0.05:^ **^ *P*<0.01.

## Abbreviations

G3BP1: Ras-GTPase-activating protein (GAP)-binding protein 1
NPC: nasopharyngeal carcinoma
EBV: Epstein-Barr virus
SG: stress granule
NSCLC: non-small cell lung cancer
EMT: epithelial-mesenchymal transition
RBP: RNA binding protein
DENR: re-initiation and release factor
EGCG: Epigallocatechin gallate
IHC: immunohistochemistry
TCGA: The Cancer Genome Atlas
GEO: Gene Expression Omnibus
STR: short tandem repeat
RIP: RNA immunoprecipitation
GAPDH: Glyceraldehyde-3-phosphate dehydrogenase
FISH: Fluorescence *in situ* hybridization
qPCR: quantitative polymerase chain reaction
cGAS: cyclic GMP-AMP synthase
PMP22: peripheral myelin protein 22
NTF2: nuclear transporter factor 2
RRM: RNA-recognition module
RGG: arginine-glycine-glycine
rasGAP: Ras GTPase Activating Protein
VF: virus factory
MRV: mammalian orthoreovirus
rG4: RNA guanine quadruplexes
uORF: upstream open reading frame
Arid5a: AT-rich interactive domain-containing protein 5a
