# Supplementary material for: G3BP1 Interact with JAK2 mRNA to Promote the Malignant Progression of Nasopharyngeal Carcinoma via Activating JAK2/STAT3 Signaling Pathway
Source: Int J Biol Sci. 2024 Jan 1;20(1):94–112. doi: 10.7150/ijbs.85341 (PMC10750281; doi:10.7150/ijbs.85341)
Supplement: Supplementary file 1 — Supplementary figures and tables. [file ijbsv20p0094s1.pdf]

Supplementary materials

Table Supplementary 1：The antibodies of WB applied in this study

Antibody	Catalog	Company
G3BP1	sc-365338	SANTA CRUZ
JAK2	ab108596	Abcam
p-STAT3	ab76315	Abcam
STAT3	A1192	Abclonal
JAK1	66466-1-Ig	Proteintech
TYK2	A2128	Abclonal
Mcl-1	#4572	CST
Bim	A19702	Abclonal
Cleaved-PARP	380374	ZEN-BIOSCIENCE
PARP	A19596	Abclonal
CDK4	11026-1-AP	Proteintech
CDK6	14052-1-AP	Proteintech
Vim	BF8006	Affinity
E-Cad	20874-1-AP	Proteintech
N-Cad	22018-1-AP	Proteintech
Ki-67	MAB-0672	MXB Biotechnologies
p-Akt (S473)	ab81283	Abcam
Akt	#9272	Cell Signaling Technology
p-mTOR (Ser2448)	#2976	Cell Signaling Technology
mTOR	66888-1-Ig	Proteintech
p-S6 (Ser240/244)	#5364	Cell Signaling Technology
S6	66886-1-Ig	Proteintech

Table Supplementary 2: The primers applied in this study

**Genes**	Primers（5’-3’）
G3BP1	F: ATGGTGATGGAGAAGCCTAGTC
	R: TCACTGCCGTGGCGCAAG
JAK1	F: CCCCCATTGATCGTCCACAA
	R: CACATACATCCCCTCCTCGC
JAK2	F: CGATCTGTGTAGCCGGTTT
	R: TCTGTCATCGTAAGGCAGGC
TYK2	F: GACAGTCCATGAGAAGTACCAAGG
	R: CTCTAGACAGGAGTAAGGCACAC
GAPDH	F: CGAGATCCCTCCAAAATCAA
	R: TTCACACCCATGACGAACAT

Table Supplementary 3: The primers in RIP assay

Primers	sequences（5’-3’）
Primer 3	F: TTGAGAAGACGGTGTGGCC
	R: CTCAGCTCCCACTCACATCC
Primer 16.17	F: CCTCGTTGGTATTGCAGTGG
	R: AGTCTTGGATCTTTGCTCGAA
Primer 41	F: TGCCAAAGGACATTCTTCAGGA
	R: ACTGTTAAGATCTCGTATGATGGCT
Primer 60	F: TGGATCAAATAAGGGATAACATGGC
	R: TGCCAGACAAGAGTGATGTT

Table Supplementary 4. Clinicopathological features of patients with NPC and non-cancerous nasopharyngeal epithelial tissues

Patients features	No. of patients (%)
**NPC patients**	
**Gender**	
Male	237 (73.1)
Female	87 (26.9)
**Age**	
<40	57 (17.6)
≥40	267 (82.4)
**T stage**	
T1	33 (10.2)
T2	132 (40.7)
T3	94 (29.0)
T4	65 (20.1)
**N stage**	
N0	36 (11.1)
N1/N2/N3	288 (88.9)
**M stage**	
M0	321 (99.1)
M1	3 (0.9)
**Survival status**	
Alive	243 (75.0)
Dead	81 (25.0)
**Clinical stages**	
I	1 (0.3)
II	64 (19.8)
III	152 (46.9)
IV	107 (33.0)
**Non-cancerous nasopharyngeal epithelium**	
**Gender**	
Male	32 (60.4)
Female	21 (39.6)
**Age**	
<40	22 (41.5)
≥40	31 (58.5)

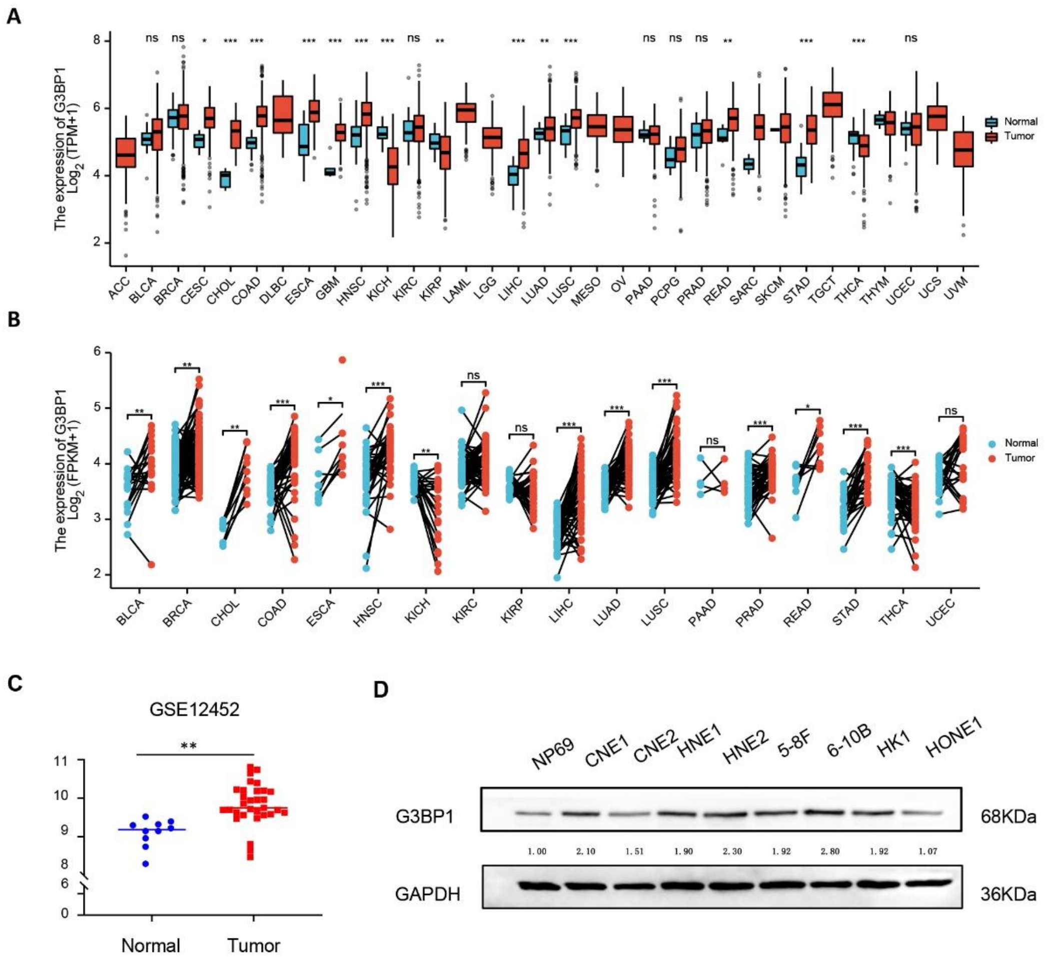

Zhan et al. Figure Supplementary 1

Figure Supplementary 1: G3BP1 was significantly overexpressed in multiple types of malignant tumors, including NPC. (A) Transcriptional expression of G3BP1 in 33 types of cancer and corresponding tissues (unpairwise samples, TCGA database). (B) Transcriptional expression of G3BP1 in 18 pairwise samples corresponding tissues (TCGA database). (C) Transcriptional expression of G3BP1 in NPC tissues and corresponding normal tissues (GEO database, GSE 12452). (D) Immortalized nasopharyngeal epithelial cell line, the basal level of G3BP1 in NPC cell lines (CNE1, CNE2, HNE1, HNE2, 5-8F, 6-10B, HK1 and HONE1) and immortalized nasopharyngeal epithelial cell line (NP69).

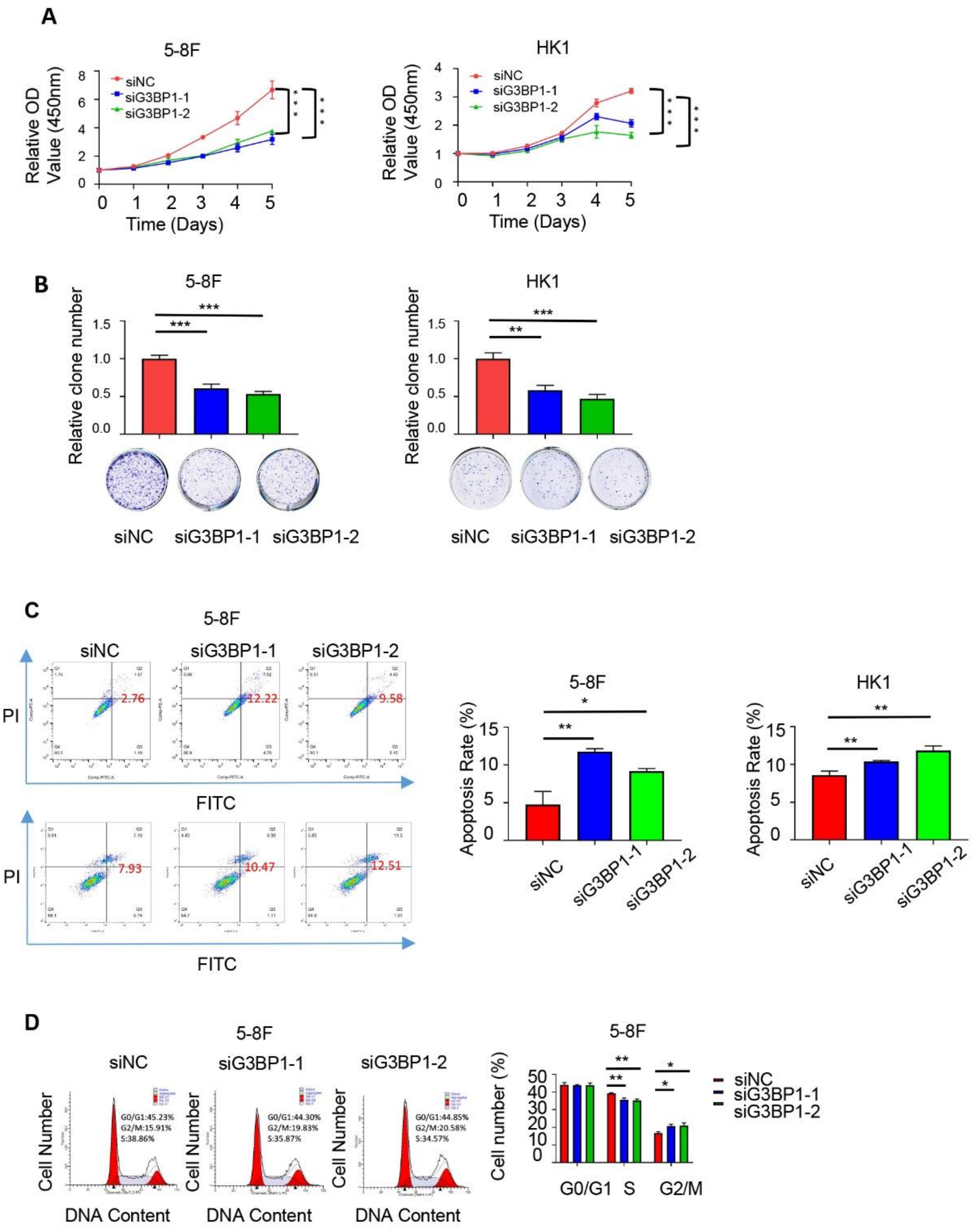

Zhan et al. Figure Supplementary 2

Figure Supplementary 2: G3BP1 promoted cell growth of NPC cells. (A and B) Knockdown of G3BP1 significantly inhibited the cell growth of 5-8F and HK1 cells (CCK8 assay and clone formation respectively). (C) Knockdown of G3BP1 significantly increased the apoptosis rate of 5-8F and HK1 cells in front of the starvation induction. (D) Cell cycle was arrested at G2/M phase under the condition of knocking-down of G3BP1 in of 5-8F cells

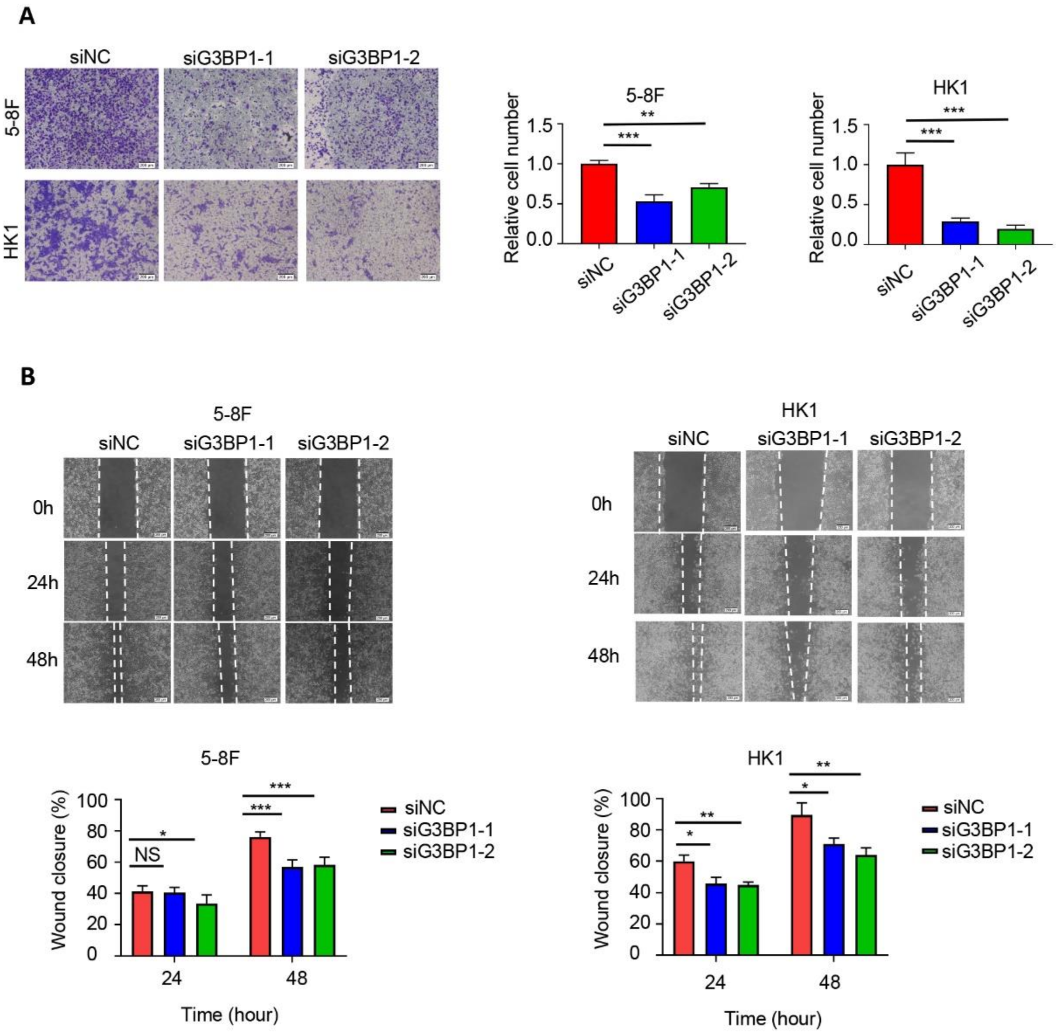

Zhan et al. Figure Supplementary 3

Figure Supplementary 3: G3BP1 promoted cell migration and invasion of NPC cells. (A) 5-8F and HK1 cells with G3BP1 knock-down showed significantly lower invasion rates in matrigel invasion assay compared with control cells. (B) 5-8F and HK1 cells with G3BP1 knock-down showed significantly lower migration rates by wound healing assay.

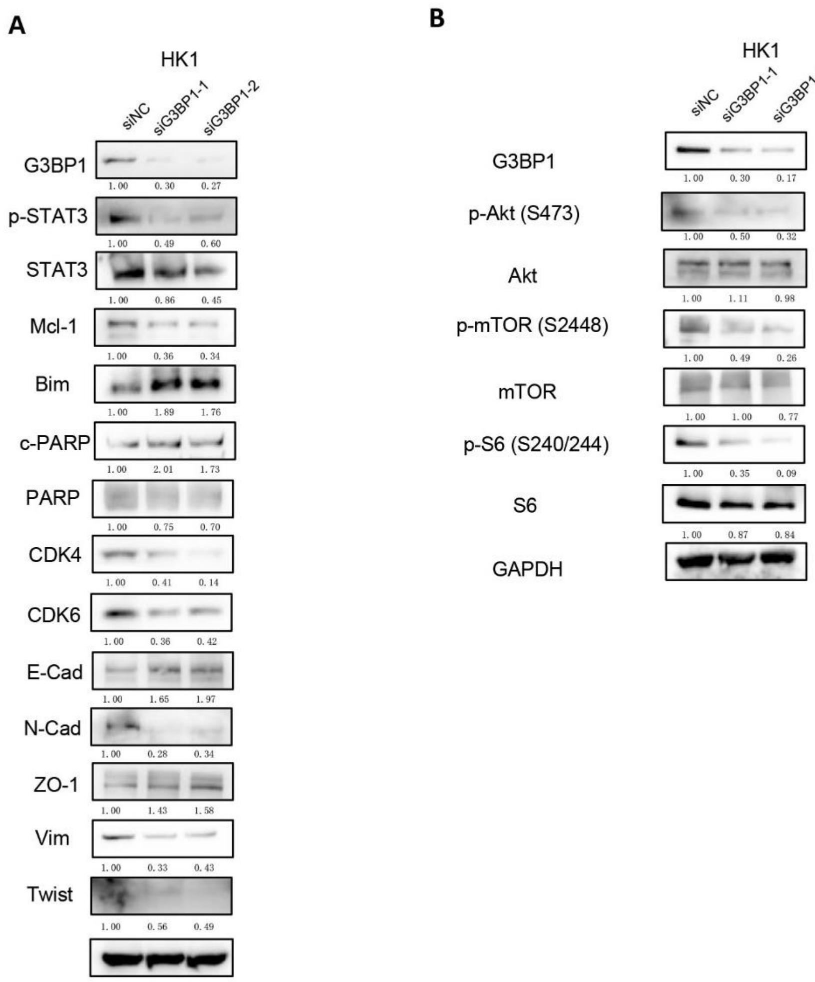

Zhan et al. Figure Supplementary 4

Figure Supplementary 4: G3BP1 activated JAK2/STAT3 and PI3K/Akt signaling pathway. G3BP1 activated JAK2/STAT3 signaling pathway in HK1 cells. (B) Knockdown G3BP1 inhibited PI3K/Akt signaling pathway in HK1 cells.

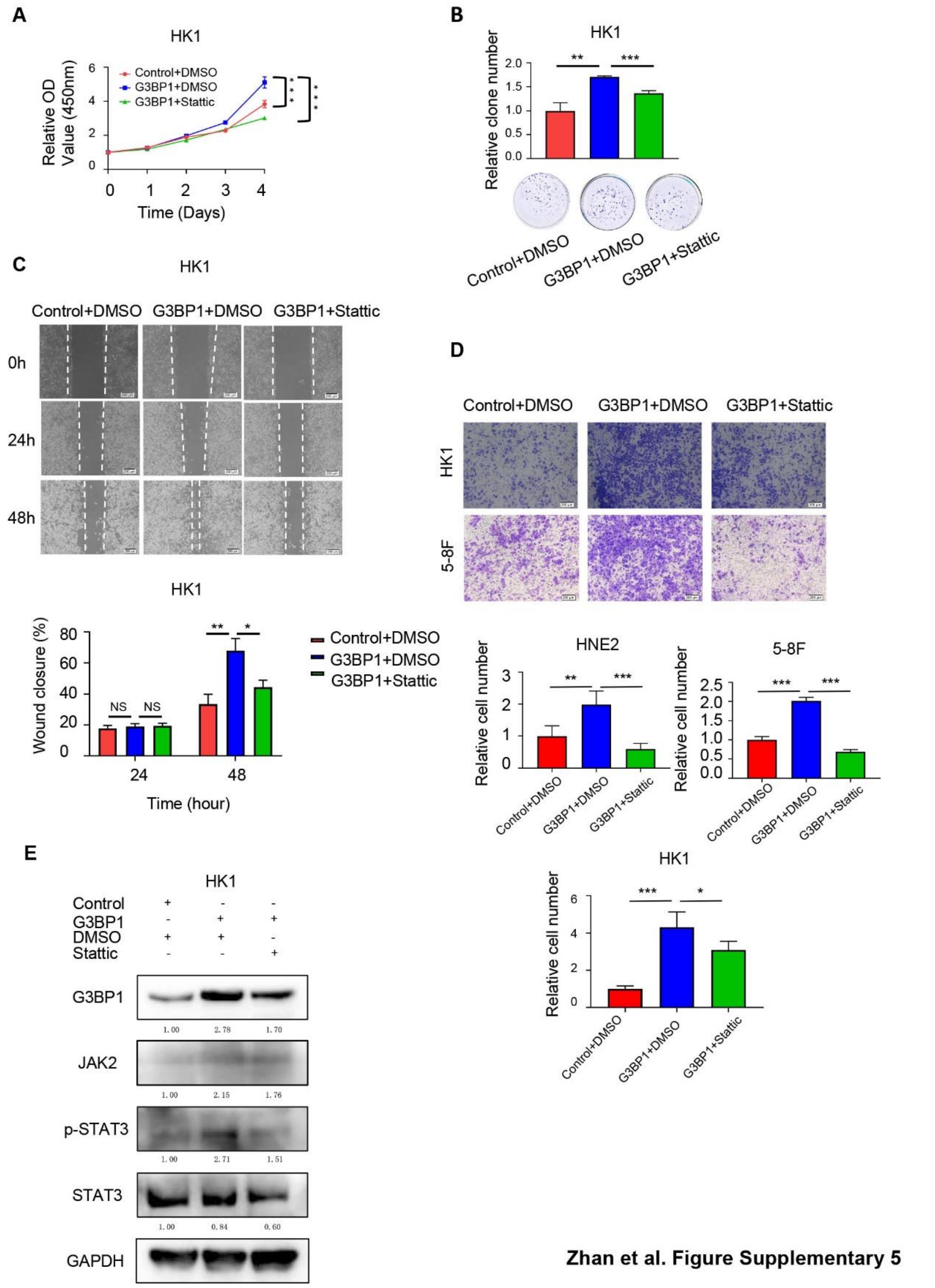

Figure Supplementary 5: G3BP1 promoted cell proliferation, migration, and invasion via JAK2/STAT3 signaling pathway. (A and B) Stattic, the inhibitor of STAT3, was used to recover the cell growth of HK1 with overexpression of G3BP1 (CCK8 assay and clone formation). (C and D) Stattic might recover the migration and invasion rates of HNE2, 5-8F and HK1 with overexpression of G3BP1. (E) p-STAT3 was up-regulated by overexpression of G3BP1, which could be recovered by stattic.

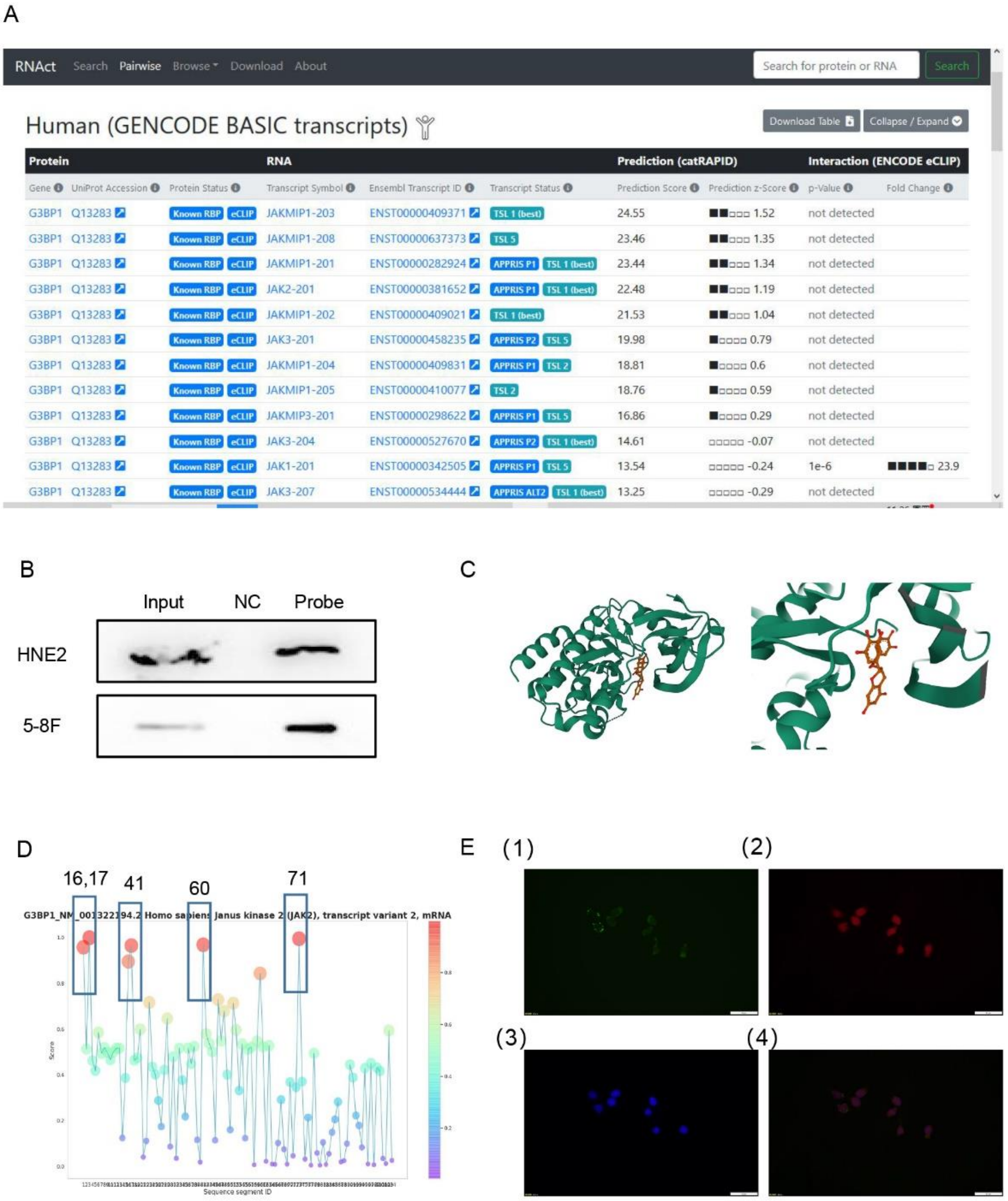

Zhan et al. Figure Supplementary 6

Figure Supplementary 6: G3BP1 was identified as an RNA binding protein that interacts with JAK2 mRNA. (A) G3BP1 may have potential to interact with three JAKs mRNA (RNAct database). (B) The binding of G3BP1 protein and JAK2 mRNA was validated RNA pulldown assay. (C) EGCG could bind to G3BP1 through visible hydrogen binding and strong electrostatic interaction by Autodock Vina V1.2.2. (D) It was suggested that the potential binding sites of G3BP1 protein and JAK2 mRNA by RBPsuite database. (E) The interaction between G3BP1 protein and JAK2 mRNA was independent of SG formation. Note: (1) The typical image of G3BP1 protein via immunofluorescence, the highlight spots were SGs. (2) The typical image of JAK2 mRNA via fluorescence in situ hybridization. (3) The typical image of DAPI. (4) The merge picture of the location of G3BP1 protein, JAK2 mRNA and DAPI.

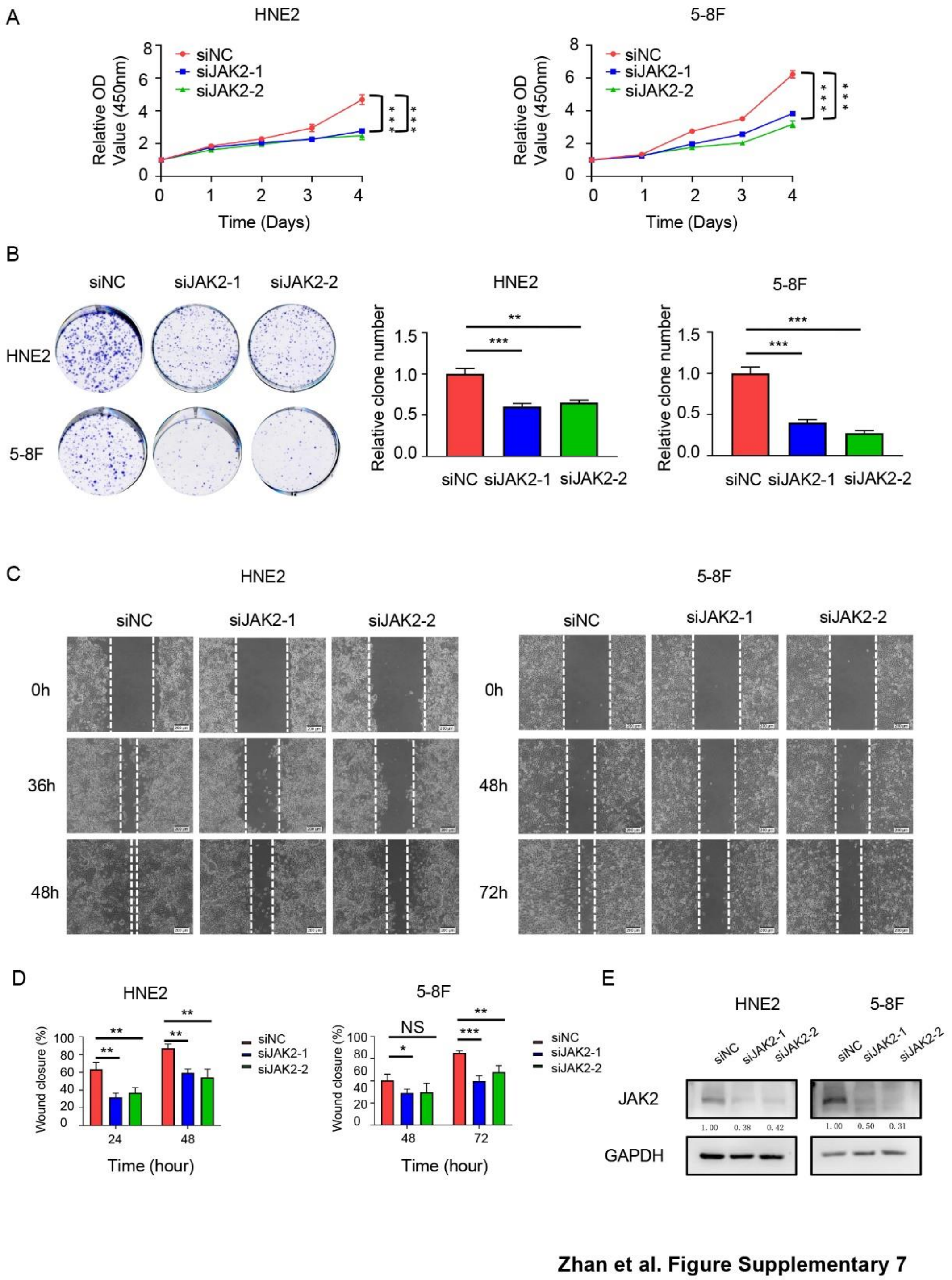

Figure Supplementary 7: JAK2 was identified as an oncogenetic factor in NPC cells. (A and B) Knockdown of JAK2 significantly inhibited the cell growth of HNE2 and 5-8F cells (CCK8 assay and clone formation respectively). (C and D) HNE2 and 5-8F cells with G3BP1 knock-down showed significantly lower migration rates by wound healing assay. (E) Two siRNAs were designed and verified in HNE2 and 5-8F cells.

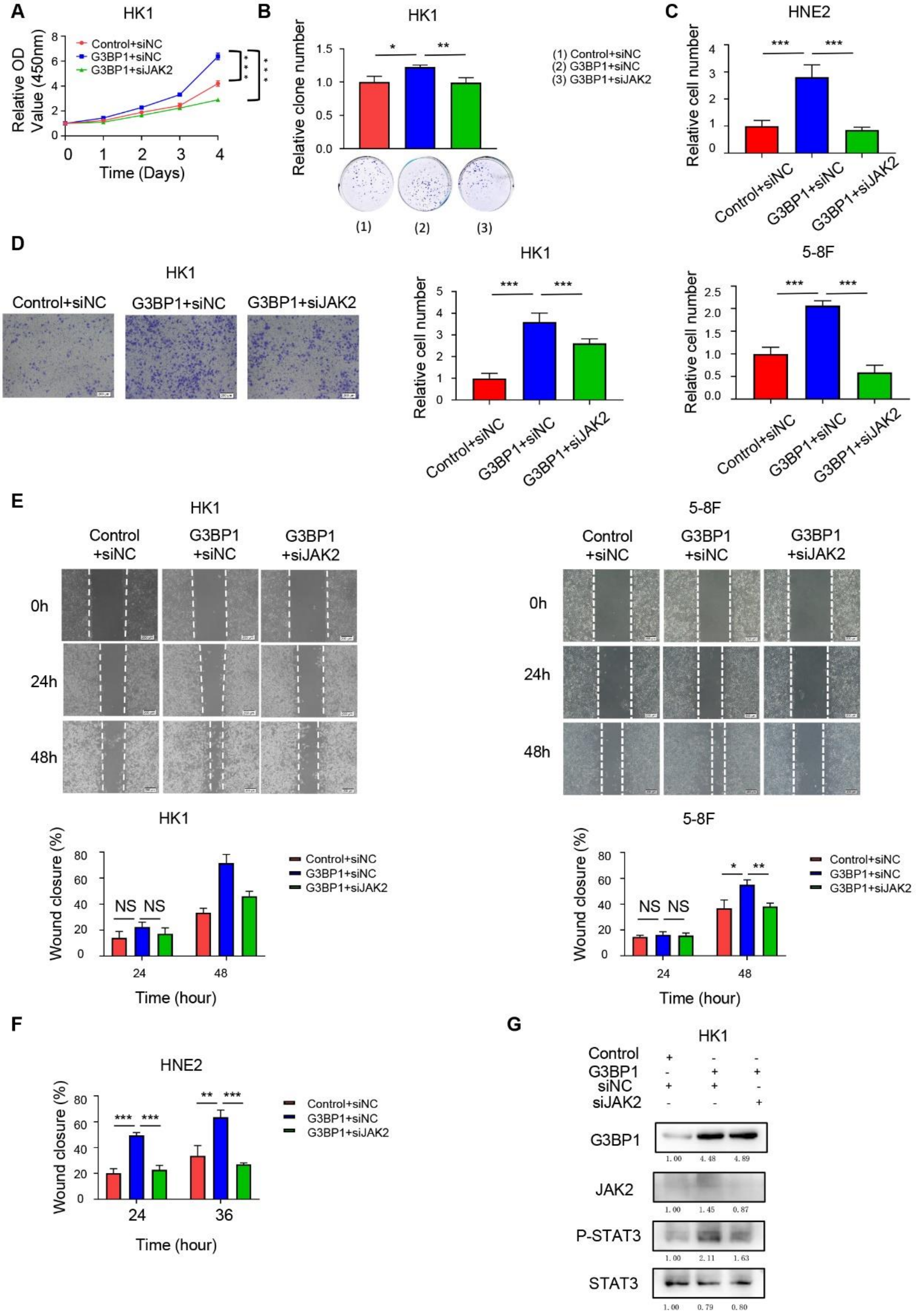

Zhan et al. Figure Supplementary 8

Figure Supplementary 8: JAK2 recovered the effect of G3BP1 in NPC cells. (A and B) knocking down JAK2 might reverse the cell growth of HK1 with overexpression of G3BP1 by CCK8 assay and clone formation. (C-D) Knocking down JAK2 might reverse the invasion rates in matrigel invasion assay of HNE2, 5-8F and HK1 with overexpression of G3BP1. (E-F) Knocking down JAK2 might reverse the migration rates in wounding healing assay of HNE2, 5-8F and HK1 with overexpression of G3BP1. (G) p-STAT3 was up-regulated by overexpression of G3BP1, which could be recovered by knock-down of JAK2.

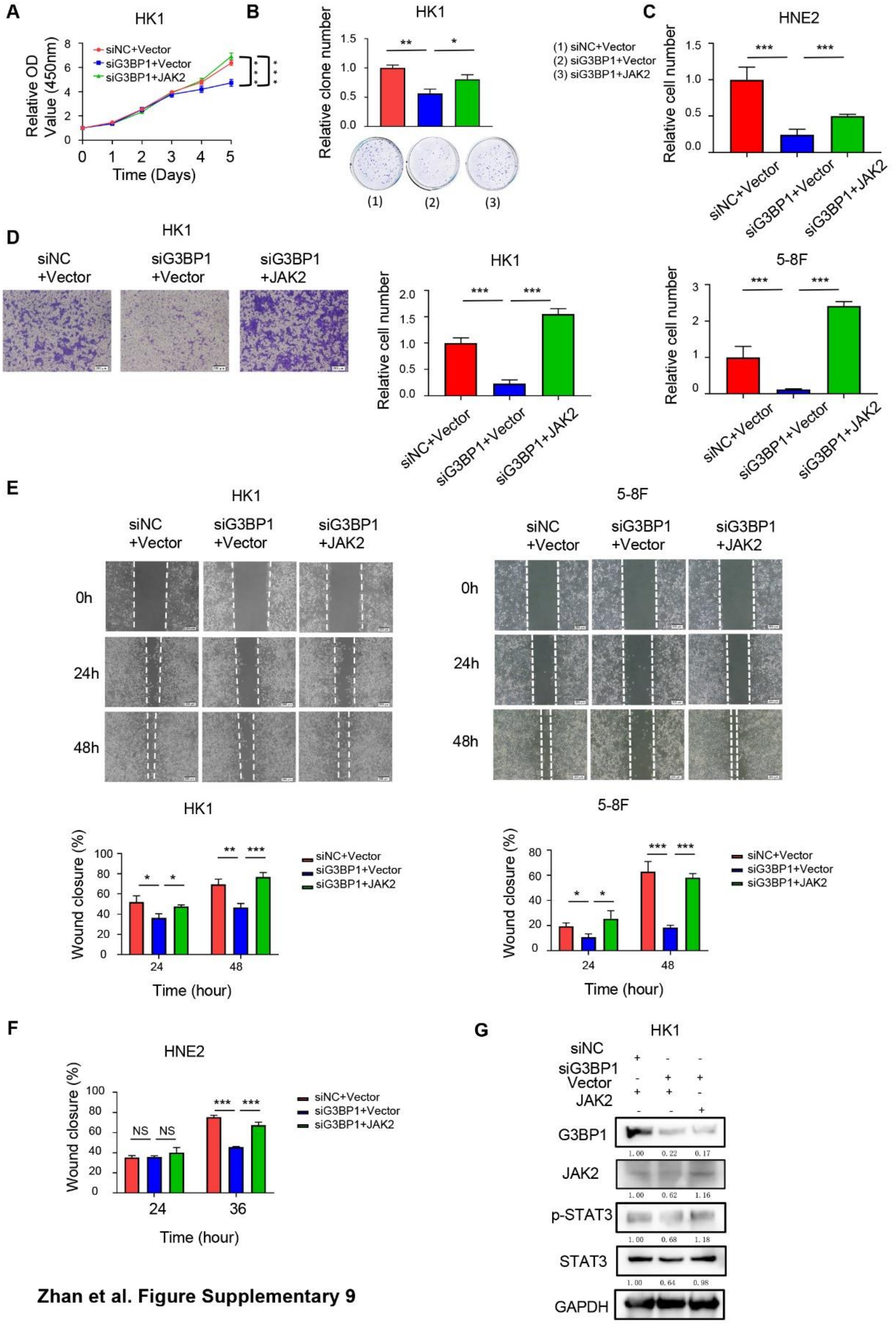

Figure Supplementary 9: JAK2 rescued the effect of G3BP1 in NPC cells. (A and B) Overexpression of JAK2 might reverse the cell growth of HK1 with knockdown of G3BP1 by CCK8 assay and clone formation. (C-D) Overexpression of JAK2 might reverse the invasion rates in matrigel invasion assay of HNE2, 5-8F and HK1 with knocking down G3BP1. (E-F) Overexpression of JAK2 might reverse the migration rates in wound healing assay of HNE2, 5-8F

and HK1 with knocking down G3BP1. (F) Knocking down G3BP1 could decrease the expression of p-STAT3, which might be recovered by overexpression of JAK2.

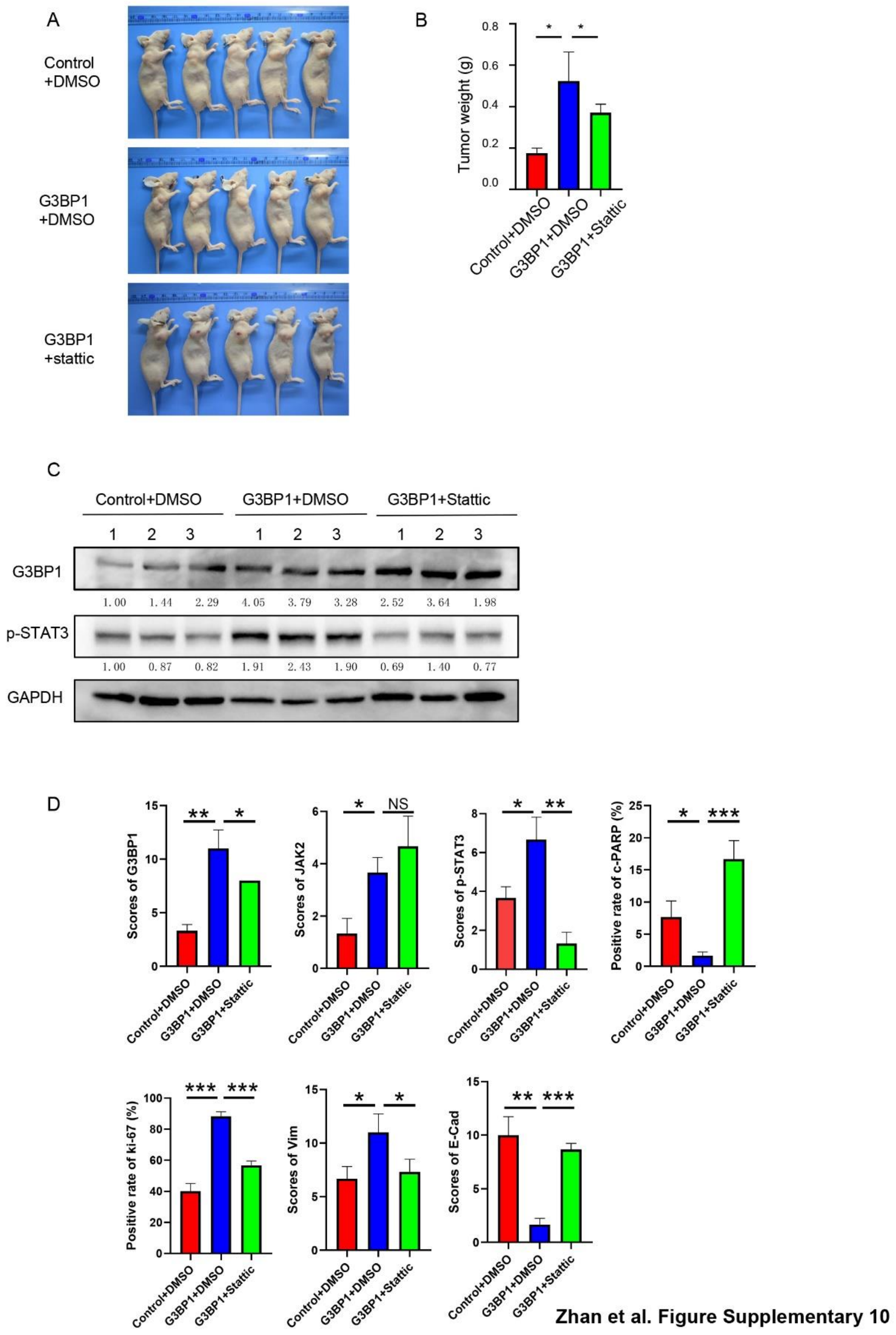

Figure Supplementary 10: G3BP1 promoted cell proliferation, and this effect could be reversed by stattic *in vivo*. (A and B) Mice image and tumor weight of nude mice with 5-8F cells in xenograft model. (C) Expression of G3BP1 and p-STAT3 proteins in mashed tumor tissues of nude mice. (D) Statistical analysis of Figure 5D.

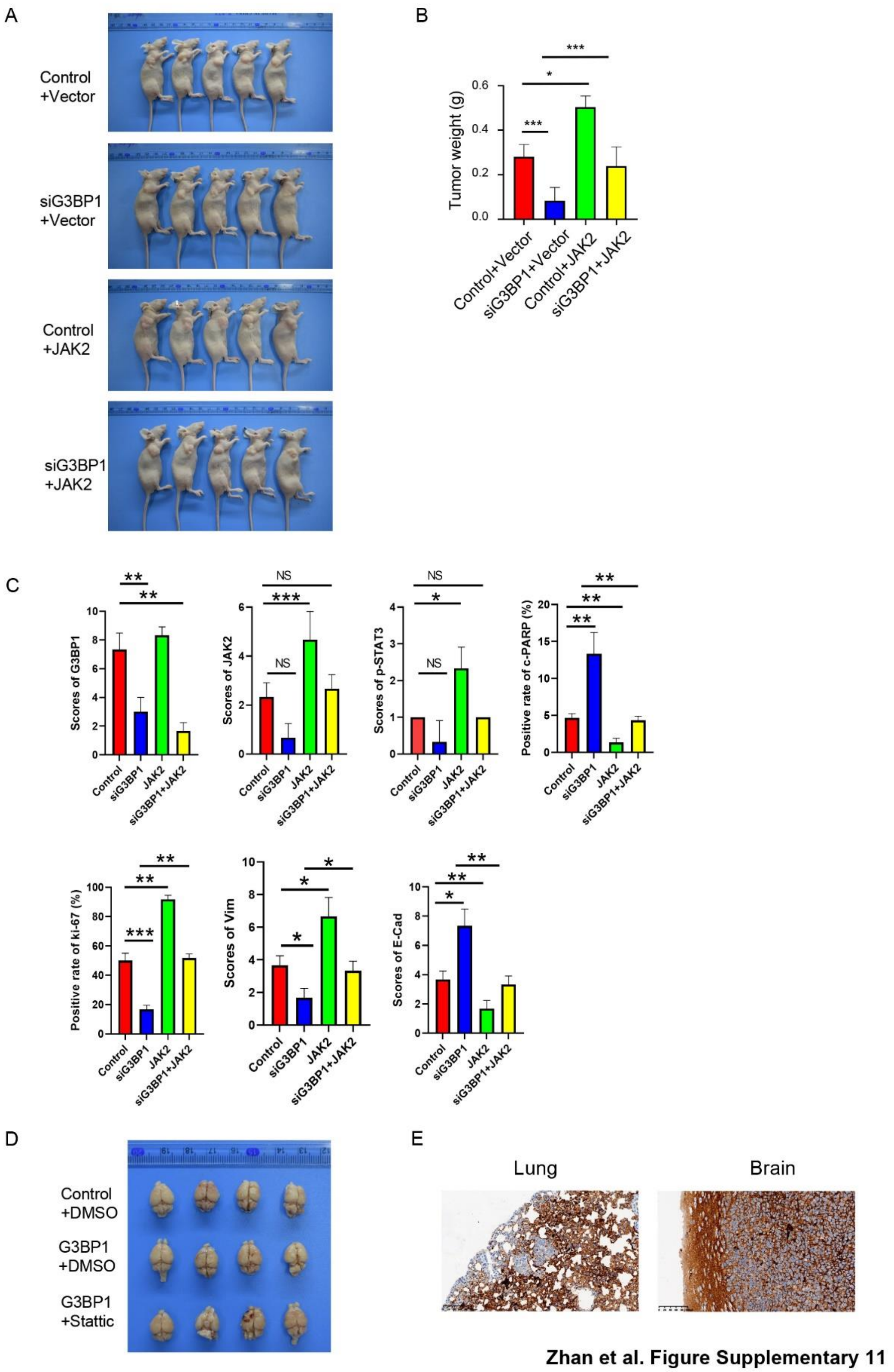

Zhan et al. Figure Supplementary 11

Figure Supplementary 11: G3BP1 promoted cell proliferation and increased metastasis in vivo. (A and B) Mice image and tumor weight of nude mice with 5-8F cells in xenograft model. (C) Statistical analysis of Figure 5G. (D) The effects of G3BP1 on the metastasis of brain *in vivo* were confirmed by tail vein metastasis model, and stattic was injected for rescue assay. (E) Representative image of IHC staining of CK in lung and brain tissues.
